# Dietary Methyl-Group Donor Intake and Breast Cancer Risk in the European Prospective Investigation into Cancer and Nutrition (EPIC)

**DOI:** 10.3390/nu13061843

**Published:** 2021-05-28

**Authors:** Heleen Van Puyvelde, Nikos Papadimitriou, Joanna Clasen, David Muller, Carine Biessy, Pietro Ferrari, Jytte Halkjær, Kim Overvad, Anne Tjønneland, Renée T. Fortner, Verena Katzke, Matthias B. Schulze, Paolo Chiodini, Giovanna Masala, Valeria Pala, Carlotta Sacerdote, Rosario Tumino, Marije F. Bakker, Antonio Agudo, Eva Ardanaz, María Dolores Chirlaque López, Maria-Jose Sánchez, Ulrika Ericson, Björn Gylling, Therese Karlsson, Jonas Manjer, Julie A. Schmidt, Geneviève Nicolas, Corinne Casagrande, Elisabete Weiderpass, Alicia K. Heath, Lode Godderis, Koen Van Herck, Dirk De Bacquer, Marc J. Gunter, Inge Huybrechts

**Affiliations:** 1 Department of Public Health and Primary Care, Faculty of Medicine and Health Sciences, Ghent University, 9000 Ghent, Belgium; heleen.vanpuyvelde@ugent.be (H.V.P.); koen.vanherck@ugent.be (K.V.H.); dirk.debacquer@ugent.bet (D.D.B.); 2 Nutrition and Metabolism Section, International Agency for Research on Cancer, CEDEX 08, 69372 Lyon, France; PapadimitriouN@fellows.iarc.fr (N.P.); biessyc@iarc.fr (C.B.); ferrarip@iarc.fr (P.F.); nicolasg@iarc.fr (G.N.); casagrandec@iarc.fr (C.C.); gunterm@iarc.fr (M.J.G.); 3 Research Foundation—Flanders (FWO), 1000 Brussels, Belgium; 4 Department of Epidemiology and Biostatistics, School of Public Health, Imperial College London, St Mary’s Hospital, London W2 1PG, UK; j.clasen18@imperial.ac.uk (J.C.); david.muller@imperial.ac.uk (D.M.); a.heath@imperial.ac.uk (A.K.H.); 5 Diet, Genes and Environment, Danish Cancer Society Research Center, DK-2100 Copenhagen, Denmark; jytteh@cancer.dk (J.H.); annet@cancer.dk (A.T.); 6 Department of Public Health, Aarhus University, DK-8000 Aarhus C, Denmark; ko@ph.au.dk; 7 Department of Public Health, University of Copenhagen, DK-2200 Copenhagen, Denmark; 8 Division of Cancer Epidemiology, German Cancer Research Center, 69120 Heidelberg, Germany; r.fortner@dkfz-heidelberg.de (R.T.F.); v.katzke@dkfz-heidelberg.de (V.K.); 9 Department of Molecular Epidemiology, German Institute of Human Nutrition Potsdam-Rehbruecke, 14558 Nuthetal, Germany; mschulze@dife.de; 10 Institute of Nutritional Science, University of Potsdam, 14558 Nuthetal, Germany; 11 Department of Mental and Physical Health and Preventive Medicine, University of Campania “Luigi Vanvitelli”, 80138 Naples, Italy; paolo.chiodini@unicampania.it; 12 Cancer Risk Factors and Life-Style Epidemiology Unit, Institute for Cancer Research, Prevention and Clinical Network—ISPRO, 50139 Firenze, Italy; g.masala@ispro.toscana.it; 13 Epidemiology and Prevention Unit, Fondazione IRCCS Istituto Nazionale dei Tumori di Milano Via Venezian, 1, 20133 Milano, Italy; valeria.pala@istitutotumori.mi.it; 14 Unit of Cancer Epidemiology, Città della Salute e della Scienza University-Hospital, 10126 Turin, Italy; carlotta.sacerdote@cpo.it; 15 Cancer Registry and Histopathology Department, Provincial Health Authority (ASP 7), 97100 Ragusa, Italy; rtuminomail@gmail.com; 16 Julius Center for Health Sciences and Primary Care, University Medical Center Utrecht, Utrecht University, 3584 CX Utrecht, The Netherlands; M.F.Bakker-8@umcutrecht.nl; 17 Cancer Epidemiology Research Program, Unit of Nutrition and Cancer, Institut Català d’Oncologa, 08908 L’Hospitalet de Llobregat, Barcelona, Spain; a.agudo@iconcologia.net; 18 Navarra Public Health Institute, 31003 Pamplona, Spain; me.ardanaz.aicua@navarra.es; 19 IdiSNA, Navarra Institute for Health Research, 31008 Pamplona, Spain; 20 CIBER Epidemiology and Public Health CIBERESP, 28029 Madrid, Spain; mdolores.chirlaque@carm.es (M.D.C.L.); mariajose.sanchez.easp@juntadeandalucia.es (M.-J.S.); 21 Department of Epidemiology, Regional Health Council, IMIB-Arrixaca, Murcia University, 30120 Murcia, Spain; 22 Escuela Andaluza de Salud Pública (EASP), 18011 Granada, Spain; 23 Instituto de Investigación Biosanitaria ibs., 18014 Granada, Spain; 24 Department of Preventive Medicine and Public Health, University of Granada, 18016 Granada, Spain; 25 Diabetes and Cardiovascular Disease, Genetic Epidemiology, Department of Clinical Sciences in Malmö, Lund University, 205 02 Malmö, Sweden; ulrika.ericson@med.lu.se; 26 Unit Pathology, Department of Medical Biosciences, Umeå Universitet, 901 85 Umeå, Sweden; bjorn.gylling@umu.se; 27 Department of Internal Medicine and Clinical Nutrition, Sahlgrenska Academy, University of Gothenburg, 405 30 Gothenburg, Sweden; therese.karlsson@gu.se; 28 Department of Surgery, Skåne University Hospital Malmö, Lund University, Bröstmottagningen, 214 28 Malmö, Sweden; jonas.manjer@med.lu.se; 29 Cancer Epidemiology Unit, Nuffield Department of Population Health, University of Oxford, Oxford OX3 LF7, UK; julie.schmidt@ndph.ox.ac.uk; 30 Office of the Director, International Agency for Research on Cancer, CEDEX 08, 69372 Lyon, France; director@iarc.fr; 31 Department of Public Health and Primary Care, KU Leuven, 3000 Leuven, Belgium; lode.godderis@kuleuven.be; 32 IDEWE (Externe dienst voor Preventie en Bescherming op het Werk), 3001 Heverlee, Belgium

**Keywords:** breast cancer, folate, choline, betaine, methionine, EPIC

## Abstract

(1) Background: Methyl-group donors (MGDs), including folate, choline, betaine, and methionine, may influence breast cancer (BC) risk through their role in one-carbon metabolism; (2) Methods: We studied the relationship between dietary intakes of MGDs and BC risk, adopting data from the European Prospective Investigation into Cancer and Nutrition (EPIC) cohort; (3) Results: 318,686 pre- and postmenopausal women were followed between enrolment in 1992–2000 and December 2013–December 2015. Dietary MGD intakes were estimated at baseline through food-frequency questionnaires. Multivariable Cox proportional hazards regression models were used to quantify the association between dietary intake of MGDs, measured both as a calculated score based on their sum and individually, and BC risk. Subgroup analyses were performed by hormone receptor status, menopausal status, and level of alcohol intake. During a mean follow-up time of 14.1 years, 13,320 women with malignant BC were identified. No associations were found between dietary intakes of the MGD score or individual MGDs and BC risk. However, a potential U-shaped relationship was observed between dietary folate intake and overall BC risk, suggesting an inverse association for intakes up to 350 µg/day compared to a reference intake of 205 µg/day. No statistically significant differences in the associations were observed by hormone receptor status, menopausal status, or level of alcohol intake; (4) Conclusions: There was no strong evidence for an association between MGDs involved in one-carbon metabolism and BC risk. However, a potential U-shaped trend was suggested for dietary folate intake and BC risk. Further research is needed to clarify this association.

## 1. Introduction

Breast cancer (BC) is a major public health concern, as the cancer with the highest incidence and the primary cause of cancer death among women worldwide [1]. In Europe, approximately 523,000 BC cases and 138,000 BC deaths were estimated in 2018 [2]. The etiology of BC is multifactorial and driven by multiple well-established risk factors including age, adult attained height, reproductive history and lactation, hormonal factors, and genetic susceptibility [3,4,5,6,7]. Additionally, various lifestyle factors such as diet, physical activity, cigarette smoking, and alcohol drinking are associated with BC, and are of particular interest because of their modifiable nature [8].

**Figure 1:**
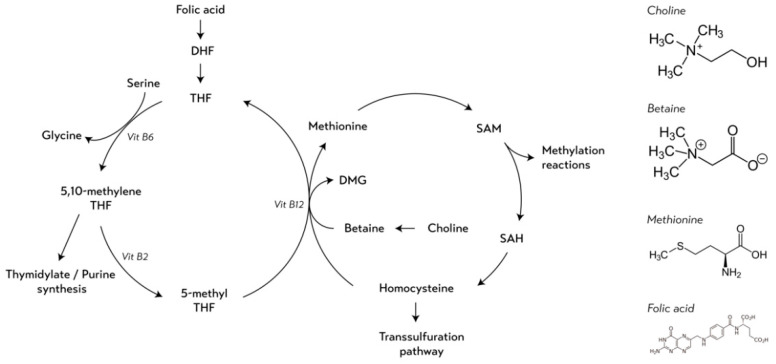
Simplified schematic overview of the one-carbon metabolism and the chemical structures of the methyl-group donors. Abbreviations: DHF: dihydrofolate; THF: tetrahydrofolate; Vit B6: vitamin B6; Vit B2: vitamin B2; Vit B12: vitamin B12; DMG: dimethylglycine; SAM: S-adenosylmethionine; SAH: S-adenosylhomocysteine.

Folate, choline, betaine, and methionine are nutrients that can be found in a variety of food sources, both plant and animal based. Foods rich in naturally occurring folates includes liver, legumes, green leafy vegetables, and cereals. The greatest amounts of choline can be found in egg yolk, meat, and fish. Beets, spinach, and grains are great sources of betaine, whereas the highest methionine concentrations can be found in protein rich foods such as meat, fish, eggs, milk, and cheese [9]. Intakes of these nutrients have been suggested to play a role in BC susceptibility through epigenetic mechanisms, such as DNA methylation [10]. These nutrients act as methyl-group donors (MGDs) within the one-carbon metabolism pathway, a metabolic network that provides ready-to-use methyl units in the form of S-adenosylmethionine (SAM) (Figure 1) [10]. SAM serves as the universal MGD responsible for all biological methylation reactions, including DNA methylation [11]. Although one carbon metabolism is under strict homeostatic regulation, severe alterations in dietary intake of MGDs may influence the availability of SAM and, therefore, may be critical for the maintenance of DNA methylation patterns. Aberrant methylation patterns may change gene expression regulation and genome stability, resulting in an altered disease risk, including altered risk of BC [10,12].

Despite folate intake being the most extensively studied nutrient among the MGDs in relation to BC risk [13,14,15,16,17,18,19,20], evidence of the association between its intake and BC remains limited and inconclusive, according to the latest update of the World Cancer Research Fund report [8]. Studies examining dietary intakes of methionine, choline, and betaine in relation to BC are less abundant [21,22]. Mainly focusing on the independent relationship of folate, choline, betaine or methionine and BC risk, few epidemiological studies take into account their biological interdependence [23,24,25,26,27]. This interdependence is a result of the junction between the folate cycle and the methionine cycle at the point where remethylation occurs, i.e., homocysteine is converted to methionine with a methyl-group donated either through betaine and choline or through the folate-derivate 5-methyl tetrahydrofolate (THF) [11] (Figure 1). Therefore, the availability and the activity of one MGD involved in one-carbon metabolism may be affected by the availability or activity of other MGDs [27,28].

In the current study, the associations of dietary MGD intakes, measured both as a calculated score based on their sum and individually, were investigated in relation to BC risk within the European Prospective Investigation into Cancer and Nutrition (EPIC), using information on usual daily alcohol consumption, menopausal status at baseline, and hormone receptor status in tumors.

## 2. Materials and Methods

### 2.1. Research Design and Study Population

The EPIC study is a large prospective cohort including over half a million men and women that was designed to study the role of diet, lifestyle, metabolic factors, and genetics in cancer incidence [29,30]. Study participants—mostly aged 35–70 years at enrolment—were recruited between 1992 and 2000 from 23 administrative centers across ten European countries: Denmark, France, Germany, Greece, Italy, Norway, Spain, Sweden, the Netherlands, and the United Kingdom. EPIC’s rationale, study design, and recruitment process have been described in detail elsewhere [29]. Approval for the EPIC study was obtained from the ethical review boards of the International Agency for Research on Cancer (IARC) and all national recruitment institutions. Written informed consent was obtained from all EPIC participants.

A final number of 318,686 women remained after exclusion from analyses based on the following criteria: participants who were lost to follow-up or whose length of follow-up was zero (*N* = 4148), participants with any cancer (except non-melanoma skin cancer) prior to baseline (*N* = 25,184), participants with missing dietary or lifestyle information (*N* = 3343), and participants in the lowest and highest 1% of the distribution of the ratio of reported total energy intake to estimated energy requirement (to avoid including extreme dietary intake values; *N* = 6723). Participants from Greece were excluded from this study (*N* = 15,233).

### 2.2. Assessment of Dietary Methyl-Group Donor Intake

Individual long-term dietary intakes were estimated at enrolment using validated country or center-specific food frequency questionnaires (FFQs), designed to capture geographically-specific diet at the individual level. Nearly all countries used self-administered FFQs, except in Spain and Italy (Naples and Ragusa) where questionnaires were administered by interviewers [29].

Dietary intakes of folate, choline, betaine, and methionine were estimated using the MGD database (MGDB) for EPIC [31]. With the exception of folate, MGDs were not added during the earlier EPIC nutrient database (ENDB) project due to the lack of food composition data in the country-specific databases [32,33,34]. Therefore, a new MGDB was compiled by matching the dietary assessment data of the EPIC cohort to four non-country-specific food composition databases (FCDB), i.e., the U.S. FCDB, Canadian FCDB, German FCDB, and Danish FCDB, using standardized operating procedures. A strong correlation (r = 0.81) was found and moderate agreement (weighted κ = 0.63) was shown between the calculated dietary folate intakes of the new MGDB and the earlier ENDB [31].

### 2.3. Lifestyle Variables and Anthropometric Measurements

Standardized baseline questionnaires were used to assemble information on sociodemographic characteristics and on a large number of lifestyle variables that are known or strongly suspected to be related to all cancer risk. Ascertainment of menopausal status at enrollment was based on a common decision tree for all countries, and is described elsewhere [20]. In addition, anthropometric measurements were collected or self-reported (in France, Oxford [UK], and Norway) at baseline [29]. Anthropometric data was adjusted to account for procedural differences between centers, and in the case of missing values, center-, age-, and sex-specific data were imputed.

### 2.4. Outcome Assessment

All BC incident cases were coded according to the International Classification of Diseases for Oncology (ICD-O-2, codes C50). Only malignant BC cases were considered incident cases for analyses. Therefore, benign or carcinoma in situ cases were right-censored at the time of diagnosis. BC incidence was identified through linkage with population cancer registries (Denmark, Italy, the Netherlands, Norway, Spain, Sweden, and the UK) or by active follow-up (France, Germany). Active follow-up included a combination of methods such as records from health insurance companies, cancer and pathology registries, as well as by direct contact with study participants or their next of kin [29]. Information on estrogen receptor (ER), progesterone receptor (PR), and human epidermal growth factor receptor (HER2) expression in tumors was collected from pathology reports provided by each EPIC center. Each cohort member was followed up for BC occurrence from the date of enrollment until the date of BC diagnosis, date of death, emigration, or end of the follow-up period (from December 2013 to December 2015, depending on the center), whichever came first.

### 2.5. Data Analysis

To summarize intakes of MGDs and to account for their overall association, an “MGD score” was generated by adding the z-scores of dietary folate, choline, betaine, and methionine intakes. Z-scores were used to scale the four MGDs because they were expressed in different units. Exposures were modelled as continuous variables and as quintiles (Q) of the MGD score, as well as individual folate, choline, betaine, and methionine intakes.

Cox proportional hazards regression models were used to estimate hazard ratios (HRs) and 95% confidence intervals (CIs) for the associations between dietary intake of MGDs at baseline (MGD score and individual components) and BC incidence. Age was used as the underlying primary time variable in the Cox models. Analyses were stratified by one-year categories of age at recruitment and by study center to account for center-specific effects such as differences in data assessment and follow-up procedures.

Cox regression models were adjusted for total energy intake (continuous, kcal/day), alcohol intake (continuous, g/day), dietary fiber intake (continuous, g/day), height (continuous, cm), BMI (continuous, kg/m^2^), highest level of education (primary/no schooling, technical/professional/secondary, longer education, unknown), physical activity (inactive, moderately inactive, moderately active, active, unknown), smoking status (never, former, current smoker, unknown), ever use of vitamin/mineral supplements (yes, no, unknown), menopausal status at recruitment (premenopausal, perimenopausal, postmenopausal (including surgical postmenopausal)), ever use of hormones for menopause (no, yes, unknown), ever use of contraceptive pill (no, yes, unknown), age at menarche (≤12, 13, 14, ≥15), and age at first full term pregnancy (nulliparous, ≤21 year, 22–29 year, ≥30 year).

To assess proportional hazards assumptions, Schoenfeld residuals were plotted against time for the dietary exposure variables (modelled continuously). No violation of the assumption was found as the residuals approached a horizontal, flat line at zero, suggesting that the coefficients did not vary over time.

To test for linear risk trends, *p* values for trend (P_trend_) were computed using the Wald test by modelling the dietary intake variables with quintile-specific medians as continuous variables. Departure from linearity was explored with restricted cubic splines using five knots, based on the likelihood ratio test (P_LRT_) comparing the linear model and the spline model using STATA IC 16 (StataCorp. 2019. College Station, TX, USA).

Associations with dietary intakes of MGDs were evaluated for total BC risk, and according to hormone receptor status (ER+, PR+, HER2+; ER−, PR−, HER2−), as well for combinations (ER+/PR+ and ER−/PR−). Statistical heterogeneity of associations across case-defined subgroups according to hormone receptor status was evaluated by calculating the I^2^ and respective *p* values for the pooled results by random-effects meta-analysis. Subgroup analyses according to menopausal status (pre- and postmenopausal (including natural and surgical) women) and alcohol intake (low: <2 drinks/week (3.4 g/day), moderate: 2–12 drinks/week (3.4–20.6 g/day), and high: >12 drinks/week (20.6 g/day); defining one drink as 12 g of alcohol) were performed. Specific interest in these subgroups is based on the difference in association in pre- and postmenopausal women and between levels of alcohol consumption, as supported by the literature [5,8,35]. Tests for interaction across non-case defined subgroups (i.e., menopausal status and level of alcohol consumption) and other a priori selected risk factors were performed by entering the product term of the MGD variable (modelled continuously and by quintiles) and the risk factor in the multivariable-adjusted model. The statistical significance of the interaction terms was assessed by comparing the difference in the log-likelihood of models with and without interaction term using the likelihood ratio test (P_interaction_). The selected risk factors included BMI at baseline (continuous, kg/m^2^), smoking status (never, former, current smoker, unknown), vitamin B12 intake (continuous, µg/day and by quintiles), vitamin B6 intake (continuous, mg/day and by quintiles), and vitamin B2 intake (continuous, mg/day and by quintiles). Moreover, tests for mutual interactions between individual MGDs (modelled continuously and as quintiles) were performed.

All analyses described above were also run excluding the first two years of follow-up after recruitment by lagging entry time for all participants, to assess the possibility of reverse causality. Additional sensitivity analyses were completed: excluding women with missing values in covariates, adjusting for additional dietary factors (e.g., co-factors involved in one-carbon metabolism), adjusting for a Mediterranean diet score or specified food groups, alternative adjustment for total or alcohol-free energy, stratifying by five-year categories of age at recruitment, and using alternative MGD scores as the exposure. All models considered sensitivity analyses can be found in the Supplementary Materials (Appendix A).

Statistical tests were two-sided and statistical significance was set at the 5% level. All analyses were performed using the SAS statistical software package version 9.4 (SAS institute, Inc., Cary, NC, USA), unless otherwise specified.

## 3. Results

Table 1 shows the distribution of the participating women in the EPIC cohort across nine countries. During a mean (±standard deviation [SD]) follow-up time of 14.1 (±3.8) years and 4,492,761 person years, a total of 13,320 malignant BC cases were reported. The median (25th percentile; 75th percentile) MGD score was −0.4 (−2.2; 1.7). The highest MGD score was observed in France (1.5 (−0.4; 3.8)) and the lowest in Germany (−2.6 (−3.8; 1.2)). The proportion of contributions of each food group to the total dietary intakes of folate, choline, betaine, and methionine can be found in Appendix B. The food sources of the MGDs were overall quite distinct. Cereal, cereal products, and vegetables were the main contributors to dietary folate and betaine intakes. Intakes of methionine and choline mainly came from dairy products, but meat, egg, and fish products were important dietary sources as well.

Baseline characteristics by lowest, third, and highest quintiles of the MGD score are reported in Appendix C. Anthropometric measures were similar across quintiles of the dietary MGD score. Women in the highest quintile were less likely to be current cigarette smokers and attained a higher level of education, but no trend was observed for physical activity. A higher MGD score was related to higher intakes of energy, alcohol, and fiber.

**Figure 2:**
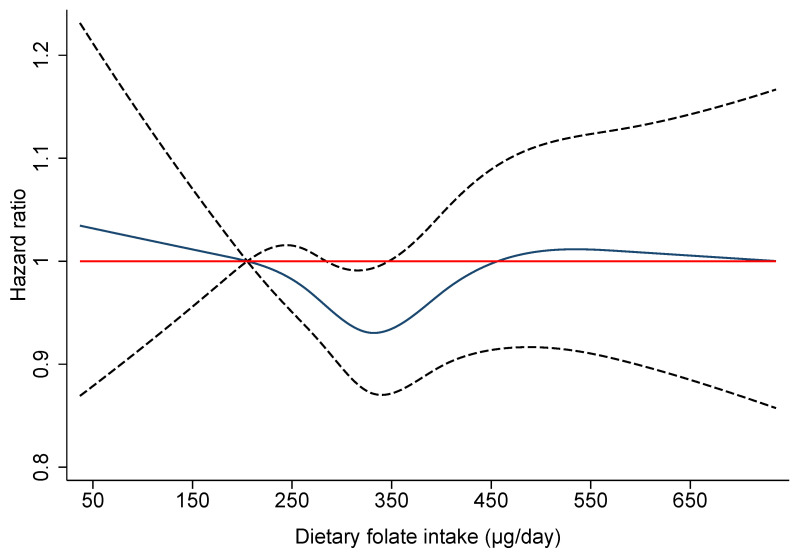
Non-linear relationship between dietary folate intake and breast cancer risk (solid line: hazard ratio; dotted line: 95% confidence intervals) among 318,686 women in the EPIC cohort, obtained by using five-knots cubic splines with the median of the first quintile as a reference value (205 µg/day). The model was stratified by study center and one year categories of age at recruitment and adjusted for total energy intake (kcal/day), alcohol intake (g/day), dietary fiber intake (g/day), height (cm), BMI (kg/m^2^), highest level of education (primary/no schooling, technical/professional/secondary, longer education, unknown), physical activity (inactive, moderately inactive, moderately active, active, unknown), smoking status (never, former, current smoker, unknown), ever use of vitamin/mineral supplements (yes, no, unknown), menopausal status at recruitment (premenopausal, postmenopausal including surgical postmenopausal, perimenopausal), age at menarche (≤12, 13, 14, ≥15), ever use of hormones for menopause (no, yes, unknown), ever use of contraceptive pill (no, yes, unknown), and age of first full term pregnancy (nulliparous, ≤21 year, 22–29 year, ≥30 year). The likelihood ratio test (P_LRT_) comparing the continuous model and the spline model: P_LRT_ folate = 0.019.

Table 2 shows the overall association of the MGD score and the individual dietary intakes of folate, choline, betaine and methionine with BC risk. The MGD score and dietary intakes of choline, betaine, and methionine showed no linear association with BC risk. However, a borderline statistically significant lower risk of overall BC was found in the third quintile of dietary folate intake compared to the first quintile with HR_Q3 vs Q1_ = 0.93 (95% CI: 0.87-1.00; *p* = 0.041). Figure 2 shows the non-linear modelling of the relation between dietary folate intake and BC risk, using 5-knot cubic splines with the 10th percentile (205 µg/day) of folate intake as the reference category. The model suggested a significant departure from linearity (P_LRT_ = 0.019), and a U-shaped trend in the association between dietary folate and BC risk was observed. A decreasing trend in BC risk was found for increasing intakes of dietary folate up to 350 µg/day, whereas the hazard ratio trended towards one for intakes greater than that value.

Subgroup analyses by menopausal status showed no associations between the MGD score or intake of the individual MGDs and BC risk (Table 2). No notable interactions were identified between dietary MGD intake and menopausal status.

Similarly, subgroup analyses by hormonal receptor status (ER−, ER+, PR−, PR+, HER2+, HER2−, ER+/PR+, or ER−/PR−) showed no significant associations, and no statistical heterogeneity was detected (all P_heterogeneity_ > 0.10) (Appendix D).

Subgroup analyses by level of alcohol consumption showed an inverse association for folate in the low alcohol consumer group in the categorical analysis (HR_Q5 vs.Q1_ = 0.85; 95% CI: 0.74-0.98; *p* = 0.028; P_trend_ = 0.052) (Table 3). However, there was no evidence that the associations between folate intakes and BC risk were different between the three groups of alcohol consumption as all the confidence intervals overlapped (moderate alcohol consumers: HR_Q5vs.Q1_ = 1.12; 95% CI: 0.96–1.31; P_trend_ = 0.059; high alcohol consumers: HR_Q5vs.Q1_ = 1.00; 95% CI: 0.78–1.29; P_trend_ = 0.998). Also, no significant interactions were found between dietary folate intake and alcohol intake (P_interaction_ = 0.231).

There was some suggestion of interaction between the MGD score, betaine intake, and methionine intake (modelled as continuous variables) and the level of alcohol consumption (P_interaction_ = 0.049; P_interaction_ = 0.037; P_interaction_ = 0.018 respectively), but estimates across groups of alcohol consumption had overlapping confidence intervals (Table 3).

There was no evidence of interaction between the MGD score, folate, choline, betaine, or methionine intakes and potential risk factors including BMI, smoking status, and intake of vitamins B2, B6, or B12 (data not shown). Furthermore, there was no suggestion of mutual interactions between individual MGDs (data not shown).

Sensitivity analyses excluding the first two years of follow-up showed overall similar results (data not shown).

## 4. Discussion

In this large-scale prospective analysis, we found no evidence of association between the MGD score, or the individual intakes of dietary folate, choline, betaine, or methionine, and BC risk. However, for dietary folate intake, a U-shaped relationship with BC risk was suggested in the overall population. In women consuming less than two alcoholic drinks per week, dietary folate intake was inversely associated with BC risk, but this association was not significantly different between groups of alcohol consumption.

The MGD score served as a measure of the overall contribution of MGD intake by adding the z-scores of dietary folate, choline, betaine, and methionine. Maruti, Ulrich, & White (2009) constructed a similar score by adding the z-scores of intakes of total folate, methionine, vitamin B2, B6, and B12 among a population of postmenopausal women [23]. Similar to our study, no significant association with BC risk was found. The overall null hazard ratios observed and the absence of statistically significant differences in the association with hormone receptor status, menopausal status, or level of alcohol intake for the MGD score, as well as for the individual intakes of MDGs, might be explained by the fact that the one-carbon metabolism is very closely regulated, and the DNA methylation rate is stabilized through several metabolites in the folate and methionine cycles, including vitamin B2, B6, and B12 [27,28]. Correspondingly, it is likely that MGD status may only affect BC risk through altered DNA methylation patterns in repeated and prolonged periods of deficient intakes. Given the variety of food sources from which MGDs can originate, both plant and animal based, deficient intakes of MGDs within the EPIC cohort are likely to be scarce.

Recently published pooled analyses of prospective cohort studies investigating the association between dietary folate intake and BC risk suggested either a J-shaped [13] or U-shaped relationship [15,16] when considering a potential non-linear relationship. Similar to the results of our study, this U-shaped relationship suggests an optimum folate intake for BC prevention. This is currently, however, not well defined. Only for a very narrow margin of dietary folate intake (from 275 µg/day to 350 µg/day) was a statistically significant lower BC risk of 5% found within the EPIC cohort. The true optimum intake likely depends on other lifestyle factors and genetic characteristics, including polymorphisms related to folate metabolism, and timing of intake. Adequate amounts and timing of dietary folate intake may prevent tumor development before the appearance of preneoplastic tissue, but overconsumption may enhance the progression of already existing tumor cells [36]. This dual role is thought to be attributable to folate’s function of providing methyl-groups for the biosynthesis of nucleotides (purines and thymidylate) required for DNA synthesis in rapidly proliferating tissues [37,38]. Given their high proliferation rate, cancer cells might also have a large demand for choline, which is a precursor for the synthesis of cell membrane phospholipids [39,40], and methionine for protein synthesis [41]; both mechanisms are essential for cell development and functioning. Therefore, the amount and timing of intake could be crucial in cancer development.

Although plausible anticancer mechanisms and explanations exist for dietary folate (including its role in the synthesis, repair, and methylation of DNA), caution is needed when interpreting the results. Even though adjustments for other B vitamins and specific food groups did not alter BC risk estimates in the current study (Appendix A), residual confounding by other unmeasured lifestyle and dietary characteristics or by other anti-carcinogenic traits of nutrients, such as beta-carotene and calcium, may have driven the observed risk pattern. Circulating concentrations could be of interest as a complementary measure to investigate mechanisms and associations between MGDs and BC risk. Within the EPIC cohort, no significant association was found between plasma folate concentrations and the risk of BC overall or by hormone receptor status [42]. Studies measuring blood levels of choline, betaine, and methionine and how they could impact BC risk are still scarce and inconclusive [43,44].

Alcohol is an acknowledged folate antagonist that negatively affects folate absorption and metabolism, resulting in lower levels of folate bioavailability in high alcohol consumers [45]. High folate intake could possibly compensate for the impaired absorption and metabolism in women with high alcohol intake. Accordingly, pooled results from epidemiological studies showed that high dietary folate intake might attenuate the higher risk of BC associated with moderate to high alcohol consumption, but a lower risk of BC for high folate intakes was not found in low alcohol consumers [15,16]. In our study, in contrast, dietary folate intake showed an inverse association with BC risk in low alcohol consumers. However, associations of folate intake with BC risk did not substantially differ between alcohol consumption groups. These results should be interpreted with caution, as residual confounding by other lifestyle factors that are highly correlated with low alcohol consumption could potentially explain the inverse association. The variation in dietary folate intakes in the EPIC cohort may not be wide enough to detect a lower BC risk among moderate or high alcohol consumers in the highest folate intake group compared to the lowest folate intake group. Furthermore, several other pathogenic mechanisms of alcohol may contribute to breast carcinogenesis and may overrule the potential inverse association between high folate intake and BC risk, including the effect of acetaldehyde and oxidative stress [46]. Alcohol also affects the one-carbon metabolism pathway through routes other than reducing folate levels; e.g., inhibition of key enzymes and cofactors (B vitamins) in the pathway and altered activity and expression of enzymes involved in DNA methylation [47]. -Further research is required to clarify the interactive effect between alcohol consumption and folate status and other B vitamins and how this relates to BC susceptibility.

This study has several strengths. First, we used an MGD score to account for the total intake of MGDs, whereas previous studies focused only on dietary intakes of single MGDs, mainly folate. Second, the association between dietary MGDs and BC risk was evaluated in the context of the EPIC study, a large prospective population-based cohort including a great amount of cancer cases with long follow-up times providing sufficient statistical power that allowed most subgroup analyses. Third, baseline information was available on a wide range of lifestyle exposures (including other dietary co-factors involved in one-carbon metabolism). Therefore, potential confounding variables could be accounted for in the analyses and a comprehensive list of sensitivity analyses could be run, allowing better interpretation of the results and confirmation of the main findings.

The main limitation of the study is the potential measurement error in the dietary intake estimation, using mainly FFQs at baseline as the only time point of exposure measurement. Any changes in dietary patterns over the follow-up time could not be considered in the analyses. Nutritional values for betaine, choline, and methionine for several foods were still lacking at the time of compilation of the MGDB (46.3%, 14.0%, and 11.8% respectively), which may have led to an underestimation of the intakes. However, missing values are expected to be logical zeros [31]. Additionally, variability in the dietary intakes of MGDs was limited, resulting in a lack of deficient or excessive intakes. Information on type and dose of supplements used was lacking, so accounting for folic acid (artificial folate) or other B vitamins from supplements was not possible. Considering these limitations in the assessment of intake of the MGDs, the absolute values reported should be interpreted with caution. The FFQs were mainly developed for ranking the EPIC participants according to dietary intakes, rather than determination of absolute values. Lastly, all presented risk estimates should also be interpreted with caution, and their biological plausibility considered, because multiple comparisons could have led to spurious findings.

## 5. Conclusions

In summary, no association was found between the intake of MGDs and BC risk in the EPIC cohort, with the exception of a U-shaped trend that was suggested for dietary folate intake and BC risk. No statistically significant differences in the associations of MGDs with BC risk were observed across levels of selected risk factors, although an inverse association was observed for dietary folate intake in the low alcohol consumption group. Prospective cohort studies on methionine, choline, and betaine are still scarce, and evidence on the association of all four MGD intakes with BC remains limited and inconclusive. Further prospective studies of dietary intakes are required to clarify the associations of the MGDs with the risk for developing different BC subtypes in different population groups (e.g., low versus high alcohol consumers), and to define optimum dietary intake ranges.

## Figures and Tables

**Table 1:** General population characteristics and dietary methyl-group donor intake: malignant breast cancer cases of women in the European Investigation into cancer and nutrition (EPIC) cohort by country.

Country	Study Participants	BC Cases	Age Range at Enrolment	Follow-Up	Person-Years	MGD Score	Dietary Folate Intake	Dietary Choline Intake	Dietary Betaine Intake	Dietary Methionine Intake
	N	N	years	years ^a^		unit ^b^	µg/day ^b^	mg/day ^b^	mg/day ^b^	g/day ^b^
Total	318,686	13,320	19.9–98.5	14.1 (3.8)	4,492,761	−0.4 (−2.2; 1.7)	327 (257; 415)	264 (214; 323)	112 (50; 174)	1.41 (1.12; 1.76)
Denmark	28,720	1869	50.1–65.8	15.1 (3.9)	432,423	−1.4 (−2.9; 0.3)	283 (235; 338)	276 (219; 345)	32 (20; 52)	1.46 (1.18; 1.79)
France	67,403	3324	41.8–71.4	12.9 (3.4)	869,372	1.5 (−0.4; 3.8)	421 (344; 510)	296 (242; 363)	136 (88; 194)	1.66 (1.33; 2.04)
Germany	27,379	817	19.9–70.1	10.4 (3.0)	284,937	−2.6 (−3.8; 1.2)	302 (252; 360)	208 (172; 251)	17 (12; 24)	1.30 (1.06; 1.58)
Italy	30,513	1211	29.1–77.8	14.3 (3.03)	434,997	0.7 (−1.4; 3.1)	340 (269; 431)	244 (202; 292)	168 (110; 249)	1.67 (1.35; 2.03)
The Netherlands	26,912	1049	20.1–70.1	14.3 (3.4)	384,249	−0.5(−1.9; 0.9)	304 (261; 352)	256 (215; 301)	146 (107; 189)	1.31 (1.08; 1.55)
Norway	33,975	1201	40.9–55.9	13.3 (2.5)	452,171	−1.0 (−2.5; 0.5)	230 (190; 275)	255 (212; 303)	135 (117; 207)	1.33 (1.09; 1.61)
Spain	24,850	655	29.0–69.8	16.0 (2.9)	398,837	0.2 (−1.4; 2.1)	359 (292; 440)	271 (224; 322)	129 (86; 178)	1.45 (1.19; 1.76)
Sweden	26,368	1314	29.2–73.6	16.8 (4.8)	442,242	−2.1 (−3.7; −0.4)	256 (207; 312)	227 (175; 288)	49 (30; 79)	1.35 (1.05; 1.67)
UK General Population	17,169	817	36.0–78.2	14.9 (4.0)	255,137	0.1 (−1.5; 1.9)	355 (293; 426)	300 (251; 352)	111 (71; 157)	1.36 (1.13; 1.62)
UK Health Conscious	35,397	1063	20.0–98.5	15.2 (3.4)	538,396	−0.04 (−1.7; 1.9)	388 (314; 476)	285 (234; 341)	130 (82; 182)	1.09 (0.82; 1.40)

^a^ Mean (SD); ^b^ Median (25th percentile; 75th percentile). Abbreviations: EPIC: European Investigation into Cancer and Nutrition; BC: breast cancer; MGD score: methyl-group donor score; N: number of participants; SD: standard deviation.

**Table 2:** Hazard ratios (HRs) and 95% confidence intervals (CIs) for breast cancer (BC) by daily dietary intake of methyl-group donors (MGDs) in 318,686 women in the EPIC cohort and by pre- and postmenopausal status.

	Overall Breast Cancer Risk (*N* = 318,686)			Premenopausal Breast Cancer Risk (*N* = 110,678)			Postmenopausal Breast Cancer Risk (*N* = 145,212)			
	BC Cases	Person Years	Multivariable Model ^a^ HR (95% CI)	BC cases	Person years	Multivariable Model ^b^ HR (95% CI)	BC Cases	Person Years	Multivariable Model ^b^ HR (95% CI)	P_interaction_^b, c^
MGD score										
Continuous (1 unit)	13,320	4,492,760	1.00 (0.98–1.01)	3314	1,592,971	0.99 (0.97–1.02)	7002	2,015,866	1.00 (0.99–1.02)	0.199^b^
Q1: ≤ −2.58	2568	885,323	1 (Ref.)	562	305,808	1 (Ref.)	1429	410,789	1 (Ref.)	
Q2: > −2.5–−1.00	2589	898,478	0.96 (0.91–1.02)	608	305,208	1.03 (0.91–1.16)	1421	420,851	0.98 (0.90–1.06)	
Q3: > −1.00–0.39	2700	905,782	0.99 (0.93–1.06)	680	320,068	1.06 (0.93–1.22)	1448	410,339	1.03 (0.94–1.12)	
Q4: > 0.39–2.31	2668	909,636	0.96 (0.89–1.04)	702	329,377	1.05 (0.90–1.22)	1352	400,920	0.98 (0.89–1.09)	
Q5: > 2.31	2795	893,541	0.98 (0.89–1.08)	762	332,509	1.05 (0.86–1.29)	1352	372,967	1.05 (0.92–1.20)	0.596^c^
P_trend_			0.857			0.675			0.449	
Dietary folate (µg/day)										
Continuous (per 1 SD = 128 µg/day)	13,320	4,492,760	1.01 (0.98–1.05)	3314	1,592,971	1.02 (0.95–1.09)	7002	2,015,866	1.02 (0.97–1.07)	0.234 ^b^
Q1: ≤ 242	2724	903,078	1 (Ref.)	568	275,216	1 (Ref.)	1470	420,708	1 (Ref.)	
Q2: > 242–298	2684	901223	0.97 (0.91–1.02)	597	296,441	0.96 (0.85–1.09)	1501	427,502	0.97 (0.90–1.05)	
Q3: > 298–357	2569	899,889	0.93 (0.87–1.00)	648	319,253	0.97 (0.85–1.11)	1412	418,308	0.94 (0.86–1.03)	
Q4: > 357–440	2683	896,336	0.98 (0.91–1.06)	739	341,300	1.04 (0.89–1.21)	1361	390,803	0.96 (0.86–1.06)	
Q5: > 440	2660	892,233	0.98 (0.89–1.08)	762	360,761	1.02 (0.84–1.23)	1258	358,544	0.96 (0.84–1.10)	0.665 ^c^
P_trend_			0.999			0.609			0.618	
Choline (mg/day)										
Continuous (per 1 SD = 90 mg/day)	13,320	4,492,760	1.00 (0.98–1.03)	3314	1,592,971	0.99 (0.94–1.05)	7002	2,015,866	1.02 (0.98–1.05)	0.088 ^b^
Q1: ≤ 202	2448	881,386	1 (Ref.)	576	320,398	1 (Ref.)	1297	392,442	1 (Ref.)	
Q2: < 202–244	2626	893,846	1.01 (0.96–1.07)	710	320,444	1.15 (1.02–1.29)	1350	401,919	0.99 (0.92–1.08)	
Q3: < 244–285	2554	904,412	0.96 (0.90–1.02)	625	326,142	0.98 (0.86–1.11)	1364	402,297	0.99 (0.91–1.08)	
Q4: < 285–339	2716	909,864	0.98 (0.92–1.05)	698	326,899	1.07 (0.93–1.23)	1392	402,238	0.98 (0.90–1.08)	
Q5: > 339	2976	903,251	1.00 (0.93–1.08)	705	299,087	1.13 (0.96–1.33)	1599	416,970	1.03 (0.93–1.14)	0.110 ^c^
P_trend_			0.995			0.307			0.580	
Betaine (mg/day)										
Continuous (per 1 SD = 88 mg/day)	13,320	4,492,760	0.99 (0.96–1.02)	3314	1,592,971	0.99 (0.94–1.04)	7002	2,015,866	0.99 (0.96–1.03)	0.303 ^b^
Q1: ≤ 39	2783	840,795	1 (Ref.)	488	233,239	1 (Ref.)	1776	455,887	1 (Ref.)	
Q2: < 39–88	2816	936,529	0.97 (0.91–1.03)	617	302,013	0.99 (0.85–1.16)	1559	448,325	0.95 (0.88–1.03)	
Q3: < 88–132	2659	906,530	1.00 (0.94–1.08)	655	322,004	0.94 (0.80–1.12)	1352	392,397	1.02 (0.93–1.12)	
Q4: < 132–193	2642	909,235	1.01 (0.94–1.09)	808	360,323	1.04 (0.88–1.23)	1248	382,114	0.99 (0.89–1.09)	
Q5: > 193	2420	899,670	0.97 (0.89–1.05)	746	375,393	0.93 (0.78–1.12)	1067	337,143	0.98 (0.88–1.09)	0.269 ^c^
P_trend_			0.741			0.513			0.985	
Methionine (g/day)										
Continuous (per 1 g/day)	13,320	449,2760	0.97 (0.92–1.02)	3314	1,592,971	0.95 (0.85–1.06)	7002	2,015,866	0.99 (0.92–1.06)	0.199 ^b^
Q1: ≤ 1.06	2282	910,975	1 (Ref.)	661	396,925	1 (Ref.)	1168	363,005	1 (Ref.)	
Q2: < 1.06–1.30	2591	896,369	1.02 (0.96–1.08)	630	317,619	0.99 (0.89–1.12)	1362	410,524	0.99 (0.92–1.08)	
Q3: < 1.30–1.54	2731	899,018	1.03 (0.96–1.09)	646	302,725	1.00 (0.89–1.13)	1497	419,107	1.05 (0.97–1.14)	
Q4: < 1.54–1.85	2815	898,204	1.01 (0.95–1.08)	673	291,694	1.01 (0.88–1.15)	1520	421,103	1.04 (0.95–1.13)	
Q5: > 1.85	2901	888,193	0.99 (0.91–1.07)	704	284,007	0.98 (0.84–1.15)	1455	402,128	1.00 (0.90–1.11)	0.596 ^c^
P_trend_			0.572			0.889			0.896	

^a^ Multivariable model: Cox regression model stratified by study center and one year categories of age at recruitment and adjusted for total energy intake (kcal/day), alcohol intake (g/day) dietary fiber intake (g/day), height (cm), BMI (kg/m^2^), highest level of education (primary/no schooling, technical/professional/secondary, longer education, unknown), physical activity (inactive, moderately inactive, moderately active, active, unknown), smoking status (never, former, current smoker, unknown), ever use of vitamin/mineral supplements (yes, no, unknown), menopausal status at recruitment (premenopausal, postmenopausal (including surgical postmenopausal), perimenopausal), age at menarche (≤12, 13, 14, ≥15), ever use of hormones for menopause (no, yes, unknown), ever use of contraceptive pill (no, yes, unknown), and age at first full term pregnancy (nulliparous, ≤21 year, 22–29 year, ≥30 year). ^b^ Multivariable model similar to multivariable model ^c^ without adjustment for menopausal status at recruitment (premenopausal, postmenopausal (including surgical postmenopausal), perimenopausal). Abbreviations: HR: hazard ratio; CI: confidence interval; BC: breast cancer; Q: quintile; Ref: reference; MGD: methyl-group donor; N: number of participants; SD: standard deviation.

**Table 3:** Hazard ratios (HRs) and 95% confidence intervals (CIs) for breast cancer (BC) by daily dietary intake of methyl-group donors (MGDs) by levels of alcohol intake in 318,686 women in the EPIC cohort.

	Low Alcohol Intake (<2 Drinks/Week) (*N* = 157,363)			Moderate Alcohol Intake (2–12 Drinks/Week) (*N* = 124,578)			High Alcohol Intake (>12 Drinks/Week) (*N* = 36,745)			
	BC Cases	Person Years	Multivariable Model ^a^ HR (95% CI)	BC Cases	Person Years	Multivariable Model ^a^ HR (95% CI)	BC Cases	Person Years	Multivariable Model ^a^ HR (95% CI)	P_interaction_^b, c^
MGD score										
Continuous (1 unit)	5882	2,241,059	0.99 (0.97–1.01)	5448	1,748,126	1.00 (0.98–1.02)	1990	503,575	1.00 (0.97–1.03)	0.049 ^b^
Q1: ≤ −2.58	1324	504,409	1 (Ref.)	963	307,475	1 (Ref.)	281	73,439	1 (Ref.)	
Q2: > −2.58–−1.00	1175	459,463	0.92 (0.85–1.01)	1039	349,334	0.95 (0.86–1.04)	375	89,680	1.12 (0.95–1.32)	
Q3: > −1.00–0.39	1154	450,289	0.91 (0.82–1.00)	1134	362,348	1.00 (0.91–1.11)	412	93,145	1.20 (1.01–1.44)	
Q4: > 0.39–2.31	1127	433,205	0.90 (0.81–1.01)	1151	369,993	1.00 (0.89–1.12)	390	106,439	1.02 (0.83–1.24)	
Q5: > 2.31	1102	393,694	0.90 (0.78–1.04)	1161	358,976	1.02 (0.87–1.19)	532	140,871	1.10 (0.86–1.41)	0.025 ^c^
P_trend_			0.210			0.586			0.867	
Dietary folate (µg/day)										
Continuous (per 1 SD = 128 µg/day)	5882	2,241,059	0.96 (0.91–1.02)	5448	1,748,126	1.03 (0.97–1.09)	1990	503,575	1.10 (1.00–1.20)	0.063 ^b^
Q1: ≤ 242	1438	533,297	1 (Ref.)	975	298,193	1 (Ref.)	311	71,589	1 (Ref.)	
Q2: > 242–298	1211	458,111	0.93 (0.86–1.02)	1076	350,013	0.98 (0.89–1.08)	397	93,099	1.02 (0.87–1.19)	
Q3: > 298–357	1067	431,110	0.85 (0.77–0.94)	1113	366,994	1.02 (0.92–1.13)	389	101,785	0.95 (0.80–1.13)	
Q4: > 357–440	1101	417,655	0.89 (0.79–0.99)	1152	368,818	1.09 (0.97–1.23)	430	109,863	1.00 (0.82–1.22)	
Q5: > 440	1065	400,886	0.85 (0.74–0.98)	1132	364,109	1.12 (0.96–1.31)	463	127,239	1.00 (0.78–1.29)	0.231 ^c^
P_trend_			0.052			0.059			0.998	
Dietary choline (mg/day)										
Continuous (per 1 SD = 90 mg/day)	5882	2,241,059	0.99 (0.95–1.02)	5448	174,8126	1.01 (0.97–1.05)	1990	503,575	1.01 (0.95–1.07)	0.064 ^b^
Q1: ≤ 202	1365	535,685	1 (Ref.)	858	284,036	1 (Ref.)	225	61,666	1 (Ref.)	
Q2: < 202–244	1226	471,093	0.97 (0.90–1.06)	1055	337,077	1.03 (0.94–1.13)	345	85,676	1.12 (0.94–1.33)	
Q3: < 244–285	1136	444,750	0.94 (0.86–1.03)	1038	359,764	0.94 (0.85–1.03)	380	99,898	1.06 (0.89–1.27)	
Q4: < 285–339	1106	418,570	0.94 (0.85–1.04)	1171	378,671	0.98 (0.88–1.09)	439	112,623	1.09 (0.91–1.31)	
Q5: > 339	1049	370,961	0.92 (0.82–1.04)	1326	388,578	1.04 (0.92–1.17)	601	143,712	1.13 (0.93–1.38)	0.292 ^c^
P_trend_			0.177			0.577			0.344	
Dietary betaine (mg/day)										
Continuous (per 1 SD = 88 mg /day)	5882	2,241,059	1.01 (0.97–1.04)	5448	1,748,126	0.96 (0.92–1.00)	1990	503,575	1.02 (0.95–1.08)	0.037 ^b^
Q1: ≤ 39	1089	378,757	1 (Ref.)	1254	359,896	1 (Ref.)	440	102,142	1 (Ref.)	
Q2: < 39–88	1160	464,885	0.93 (0.85–1.03)	1240	373,502	0.96 (0.87–1.05)	416	98,141	1.06 (0.91–1.24)	
Q3: < 88–132	1264	474,910	1.01 (0.91–1.13)	1031	342,637	0.92 (0.83–1.03)	364	88,983	1.19 (0.99–1.44)	
Q4: < 132–193	1194	447,685	1.03 (0.92–1.15)	1040	353,835	0.94 (0.83–1.05)	408	107,715	1.13 (0.93–1.38)	
Q5: > 193	1175	474,821	0.98 (0.87–1.10)	883	318,255	0.89 (0.79–1.02)	362	106,593	1.12 (0.90–1.39)	0.221 ^c^
P_trend_			0.741			0.126			0.497	
Dietary methionine (g/day)										
Continuous (per 1 g/day)	5882	2,241,059	0.95 (0.88–1.02)	5448	1,748,126	1.02 (0.94–1.10)	1990	503,575	0.88 (0.77–1.00)	0.018 ^b^
Q1: ≤ 1.06	1175	510,519	1 (Ref.)	826	321,954	1 (Ref.)	281	78,502	1 (Ref.)	
Q2: < 1.06–1.30	1230	469,493	1.05 (0.96–1.14)	1033	339,137	1.01 (0.92–1.11)	328	87,739	0.94 (0.80–1.11)	
Q3: < 1.30–1.54	1220	451,112	1.03 (0.95–1.13)	1093	353,461	0.99 (0.89–1.09)	418	94,445	1.09 (0.92–1.28)	
Q4: < 1.54–1.85	1140	423,892	0.99 (0.90–1.09)	1226	367,949	1.02 (0.9–1.14)	449	106,363	1.02 (0.86–1.22)	
Q5: > 1.85	1117	386,043	0.98 (0.87–1.10)	1270	365,624	1.03 (0.91–1.16)	514	136,526	0.87 (0.71–1.07)	0.023 ^c^
P_trend_			0.460			0.581			0.200	

^a^ Multivariable model: Cox regression model stratified by study center and by one year categories of age at recruitment and adjusted for total energy intake (kcal/day), dietary fiber intake (g/day), height (cm), BMI (kg/m^2^), highest level of education (primary/no schooling, technical/professional/secondary, longer education, unknown), physical activity (inactive, moderately inactive, moderately active, active, unknown), smoking status (never, former, current smoker, unknown), ever use of vitamin/mineral supplements (yes, no, unknown), menopausal status at recruitment (premenopausal, postmenopausal (including surgical postmenopausal), perimenopausal), age at menarche (≤12, 13, 14, ≥15), ever use of hormones for menopause (no, yes, unknown), ever use of contraceptive pill (no, yes, unknown), and age at first full term pregnancy (nulliparous, ≤21 year, 22–29 year, ≥30 year). ^b^ P_interaction_ between MGDs and alcohol intake when considering the MGDs as continuous variables and alcohol intake as a categorical variable (low, moderate and high alcohol consumers). ^c^ P_interaction_ between MGDs and alcohol intake when considering the MGDs as categorical variables (quintiles) and alcohol intake as a categorical variable (low, moderate and high alcohol consumers). Abbreviations: HR: hazard ratio; CI: confidence interval; BC: breast cancer; Q: quintile; MGD: methyl-group donor; Ref: reference; N: number of participants; SD: standard deviation.

## Data Availability

EPIC data and biospecimens are available for investigators who seek to answer important questions on health and disease in the context of research projects that are consistent with the legal and ethical standard practices of IARC/WHO and the EPIC Centers. The primary responsibility for accessing the data obtained in the frame of the present publication belongs to the EPIC centers that provided them. The use of a random sample of anonymized data from the EPIC study can be requested by contacting epic@iarc.fr. The request will then be passed on to members of the EPIC Steering Committee for deliberation.

## Supplementary Materials

The following are available online at https://www.mdpi.com/article/10.3390/nu13061843/s1, Appendix A—Table S1: Hazard ratios (HRs) and 95% confidence intervals (CIs) for breast cancer (BC) by daily dietary intake of methyl-group donors in women in the EPIC cohort after different sensitivity tests; Appendix B—Table S2: Proportion of contributions of each food group to the total dietary intakes of folate, choline, betaine, and methionine among 318,686 women in the European Prospective Investigation into Cancer and Nutrition (EPIC) cohort; Appendix C—Table S3: Characteristics of women in the European Prospective Investigation into Cancer and Nutrition (EPIC) cohort (*N* = 318,686) by lowest, middle, and highest quintiles of the methyl-group donor score (MGD score); Appendix D—Table S4: Hazard ratios (HRs) and 95% confidence intervals (CIs) for breast cancer (BC) by daily dietary intake of methyl-group donors for estrogen receptor (ER) positive and negative tumors in women in the EPIC cohort; Appendix D—Table S5: Hazard ratios (HRs) and 95% confidence intervals (CIs) for breast cancer (BC) by daily dietary intake of methyl-group donors for progesterone receptor (PR) positive and negative tumors in women in the EPIC cohort; Appendix D—Table S6: Hazard ratios (HRs) and 95% confidence intervals (CIs) for breast cancer (BC) by daily dietary intake of methyl-group donors for human epidermal growth factor receptor (HER2) positive and negative tumors in women in the EPIC cohort; Appendix D—Table S7: Hazard ratios (HRs) and 95% confidence intervals (CIs) for breast cancer (BC) by daily dietary intake of methyl-group donors for estrogen receptor and progesterone receptor positive or negative tumors in women in the EPIC cohort.

## References

[1] Bray F., Ferlay J., Soerjomataram I., Siegel R.L., Torre L.A., Jemal A. (2018). Global cancer statistics 2018: Globocan estimates of incidence and mortality worldwide for 36 cancers in 185 countries. CA Cancer J. Clin..

[2] Ferlay J., Colombet M., Soerjomataram I., Dyba T., Randi G., Bettio M., Gavin A., Visser O., Bray F. (2018). Cancer incidence and mortality patterns in europe: Estimates for 40 countries and 25 major cancers in 2018. Eur. J. Cancer.

[3] Anothaisintawee T., Wiratkapun C., Lerdsitthichai P., Kasamesup V., Wongwaisayawan S., Srinakarin J., Hirunpat S., Woodtichartpreecha P., Boonlikit S., Teerawattananon Y. (2013). Risk factors of breast cancer: A systematic review and meta-analysis. Asia Pac. J. Public Health.

[4] (2002). Collaborative Group on Hormonal Factors in Breast Cancer. Breast cancer and breastfeeding: Collaborative reanalysis of individual data from 47 epidemiological studies in 30 countries, including 50,302 women with breast cancer and 96,973 women without the disease. Lancet.

[5] (2012). Collaborative Group on Hormonal Factors in Breast Cancer. Menarche, menopause, and breast cancer risk: Individual participant meta-analysis, including 118,964 women with breast cancer from 117 epidemiological studies. Lancet Oncol..

[6] (2001). Collaborative Group on Hormonal Factors in Breast Cancer. Familial breast cancer: Collaborative reanalysis of individual data from 52 epidemiological studies including 58,209 women with breast cancer and 101,986 women without the disease. Lancet.

[7] Wirén S., Häggström C., Ulmer H., Manjer J., Bjørge T., Nagel G., Johansen D., Hallmans G., Engeland A., Concin H. (2014). Pooled cohort study on height and risk of cancer and cancer death. Cancer Causes Control.

[8] WCRF (2018). Diet, Nutrition, Physical Activity and Breast Cancer.

[9] U.S. Department of Agriculture, Agricultural Research Service (2020). USDA National Nutrient Database for Standard Reference, Release 28. 2015. Documentation and User Guide. http://www.ars.usda.gov/ba/bhnrc/ndl.

[10] Friso S., Udali S., De Santis D., Choi S.W. (2017). One-carbon metabolism and epigenetics. Mol. Asp. Med..

[11] Jiménez-Chillarón J.C., Díaz R., Martínez D., Pentinat T., Ramón-Krauel M., Ribó S., Plösch T. (2012). The role of nutrition on epigenetic modifications and their implications on health. Biochimie.

[12] Anderson O.S., Sant K.E., Dolinoy D.C. (2012). Nutrition and epigenetics: An interplay of dietary methyl donors, one-carbon metabolism and DNA methylation. J. Nutr. Biochem..

[13] Zhang Y.-F., Shi W.-W., Gao H.-F., Zhou L., Hou A.-J., Zhou Y.-H. (2014). Folate intake and the risk of breast cancer: A dose-response meta-analysis of prospective studies. PLoS ONE.

[14] Liu M., Cui L.H., Ma A.G., Li N., Piao J.M. (2014). Lack of effects of dietary folate intake on risk of breast cancer: An updated meta-analysis of prospective studies. Asian Pac. J. Cancer Prev..

[15] Chen P., Li C., Li X., Li J., Chu R., Wang H. (2014). Higher dietary folate intake reduces the breast cancer risk: A systematic review and meta-analysis. Br. J. Cancer.

[16] Zeng J., Gu Y., Fu H., Liu C., Zou Y., Chang H. (2020). Association between one-carbon metabolism-related vitamins and risk of breast cancer: A systematic review and meta-analysis of prospective studies. Clin. Breast Cancer.

[17] Tio M., Andrici J., Cox M.R., Eslick G.D. (2014). Folate intake and the risk of prostate cancer: A systematic review and meta-analysis. Prostate Cancer Prostatic Dis..

[18] Lewis S.J., Harbord R.M., Harris R., Smith G.D. (2006). Meta-analyses of observational and genetic association studies of folate intakes or levels and breast cancer risk. JNCI J. Natl. Cancer Inst..

[19] Larsson S.C., Giovannucci E., Wolk A. (2007). Folate and risk of breast cancer: A meta-analysis. JNCI J. Natl. Cancer Inst..

[20] De Batlle J., Ferrari P., Chajes V., Park J.Y., Slimani N., McKenzie F., Overvad K., Roswall N., Tjonneland A., Boutron-Ruault M.C. (2015). Dietary folate intake and breast cancer risk: European prospective investigation into cancer and nutrition. J. Natl. Cancer Inst..

[21] Sun S.W., Li X., Ren A.J., Du M.L., Du H.N., Shu Y.Q., Zhu L.J., Wang W. (2016). Choline and betaine consumption lowers cancer risk: A meta-analysis of epidemiologic studies. Sci. Rep..

[22] Wu W., Kang S., Zhang D. (2013). Association of vitamin b6, vitamin b12 and methionine with risk of breast cancer: A dose-response meta-analysis. Br. J. Cancer.

[23] Maruti S.S., Ulrich C.M., White E. (2009). Folate and one-carbon metabolism nutrients from supplements and diet in relation to breast cancer risk. Am. J. Clin. Nutr..

[24] Bassett J.K., Baglietto L., Hodge A.M., Severi G., Hopper J.L., English D.R., Giles G.G. (2013). Dietary intake of b vitamins and methionine and breast cancer risk. Cancer Causes Control.

[25] Rohan T.E., Jain M.G., Howe G.R., Miller A.B. (2000). Dietary folate consumption and breast cancer risk. J. Natl. Cancer Inst..

[26] Shrubsole M.J., Shu X.O., Li H.L., Cai H., Yang G., Gao Y.T., Gao J., Zheng W. (2011). Dietary b vitamin and methionine intakes and breast cancer risk among chinese women. Am. J. Epidemiol..

[27] Kabat G., Miller A., Jain M., Rohan T. (2008). Dietary intake of selected b vitamins in relation to risk of major cancers in women. Br. J. Cancer.

[28] Nijhout H.F., Reed M.C., Anderson D.F., Mattingly J.C., James S.J., Ulrich C.M. (2006). Long-range allosteric interactions between the folate and methionine cycles stabilize DNA methylation reaction rate. Epigenetics.

[29] Riboli E., Hunt K., Slimani N., Ferrari P., Norat T., Fahey M., Charrondiere U., Hemon B., Casagrande C., Vignat J. (2002). European prospective investigation into cancer and nutrition (epic): Study populations and data collection. Public Health Nutr..

[30] Riboli E., Kaaks R. (1997). The epic project: Rationale and study design. European prospective investigation into cancer and nutrition. Int. J. Epidemiol..

[31] Van Puyvelde H., Versele V., De Backer M., Casagrande C., Nicolas G., Clasen J.L., Julián C., Skeie G., Chirlaque M.-D., Mahamat-Saleh Y. (2020). Methodological approaches to compile and validate a food composition database for methyl-group carriers in the european prospective investigation into cancer and nutrition (epic) study. Food Chem..

[32] Bouckaert K.P., Slimani N., Nicolas G., Vignat J., Wright A.J., Roe M., Witthöft C.M., Finglas P.M. (2011). Critical evaluation of folate data in european and international databases: Recommendations for standardization in international nutritional studies. Mol. Nutr. Food Res..

[33] Slimani N., Deharveng G., Unwin I., Southgate D., Vignat J., Skeie G., Salvini S., Parpinel M., Møller A., Ireland J. (2007). The epic nutrient database project (endb): A first attempt to standardize nutrient databases across the 10 european countries participating in the epic study. Eur. J. Clin. Nutr..

[34] Nicolas G., Witthöft C.M., Vignat J., Knaze V., Huybrechts I., Roe M., Finglas P., Slimani N. (2016). Compilation of a standardised international folate database for epic. Food Chem..

[35] Tjonneland A., Christensen J., Olsen A., Stripp C., Thomsen B.L., Overvad K., Peeters P.H., van Gils C.H., Bueno-de-Mesquita H.B., Ocke M.C. (2007). Alcohol intake and breast cancer risk: The european prospective investigation into cancer and nutrition (epic). Cancer Causes Control.

[36] Ulrich C.M. (2007). Folate and cancer prevention: A closer look at a complex picture. Am. J. Clin. Nutr..

[37] Xu X., Chen J. (2009). One-carbon metabolism and breast cancer: An epidemiological perspective. J. Genet. Genom..

[38] Newman A.C., Maddocks O.D.K. (2017). One-carbon metabolism in cancer. Br. J. Cancer.

[39] Cho E., Zeisel S.H., Jacques P., Selhub J., Dougherty L., Colditz G.A., Willett W.C. (2006). Dietary choline and betaine assessed by food-frequency questionnaire in relation to plasma total homocysteine concentration in the framingham offspring study. Am. J. Clin. Nutr..

[40] Zeisel S.H. (2012). Dietary choline deficiency causes DNA strand breaks and alters epigenetic marks on DNA and histones. Mutat. Res. Fundam. Mol. Mech. Mutagenesis.

[41] Cavuoto P., Fenech M.F. (2012). A review of methionine dependency and the role of methionine restriction in cancer growth control and life-span extension. Cancer Treat. Rev..

[42] Matejcic M., de Batlle J., Ricci C., Biessy C., Perrier F., Huybrechts I., Weiderpass E., Boutron-Ruault M.C., Cadeau C., His M. (2017). Biomarkers of folate and vitamin b12 and breast cancer risk: Report from the epic cohort. Int. J. Cancer.

[43] Du Y.F., Lin F.Y., Long W.Q., Luo W.P., Yan B., Xu M., Mo X.F., Zhang C.X. (2017). Serum betaine but not choline is inversely associated with breast cancer risk: A case-control study in china. Eur. J. Nutr..

[44] His M., Viallon V., Dossus L., Gicquiau A., Achaintre D., Scalbert A., Ferrari P., Romieu I., Onland-Moret N.C., Weiderpass E. (2019). Prospective analysis of circulating metabolites and breast cancer in epic. BMC Med..

[45] Scoccianti C., Lauby-Secretan B., Bello P.-Y., Chajes V., Romieu I. (2014). Female breast cancer and alcohol consumption: A review of the literature. Am. J. Prev. Med..

[46] Purohit V., Khalsa J., Serrano J. (2005). Mechanisms of alcohol-associated cancers: Introduction and summary of the symposium. Alcohol.

[47] Varela-Rey M., Woodhoo A., Martinez-Chantar M.L., Mato J.M., Lu S.C. (2013). Alcohol, DNA methylation, and cancer. Alcohol Res..

