# Supplementary material for: Dietary Methyl-Group Donor Intake and Breast Cancer Risk in the European Prospective Investigation into Cancer and Nutrition (EPIC)
Source: Nutrients. 2021 May 28;13(6):1843. doi: 10.3390/nu13061843 (PMC8228096; doi:10.3390/nu13061843)
Supplement: Supplementary file 1 [file nutrients-13-01843-s001.zip › nutrients-1210029-supplementary.pdf]

APPENDIX A - Dietary methyl-group donor intake and breast cancer risk: European Prospective Investigation into Cancer and Nutrition (EPIC)

**Appendix A - Table S1:** Hazard ratios (HRs) and 95% confidence intervals (CIs) for breast cancer (BC) by daily dietary intake of methyl-group donors in women in the EPIC cohort **after different sensitivity tests**					
**Model**	**Exposure**	**N of BC cases**	**Person years**	**Multivariable model HR (95% CI)**	**P_{cont}**
Model a	MGD score	13,320	4,492,760	1.00 (0.98-1.01)	0.456
Model a	Dietary folate (per 1 SD/day = 128 µg/day)	13,320	4,492,760	1.01 (0.98-1.05)	0.547
Model a	Choline (per 1 SD/day = 90 mg/day)	13,320	4,492,760	1.00 (0.98-1.03)	0.943
Model a	Betaine (per 1 SD/day = 88 mg/day)	13,320	4,492,760	0.99 (0.96-1.02)	0.419
Model a	Methionine (per 1g/day)	13,320	449,2760	0.97 (0.92-1.02)	0.188
Model S1	MGD score	13,320	4,492,760	1.00 (0.99-1.02)	0.832
Model S1	Dietary folate (per 1 SD/day = 128 µg/day)	13,320	4,492,760	1.02 (0.98-1.06)	0.247
Model S1	Choline (per 1 SD/day = 90 mg/day)	13,320	4,492,760	1.02 (0.99-1.05)	0.214
Model S1	Betaine (per 1 SD/day = 88 mg/day)	13,320	4,492,760	0.99 (0.96-1.01)	0.353
Model S1	Methionine (per 1g/day)	13,320	4,492,760	0.98 (0.91-1.05)	0.486
Model S2	MGD score	13,320	4,492,760	1.00 (0.98-1.01)	0.875
Model S2	Dietary folate (per 1 SD/day =128 µg/day)	13,320	4,492,760	1.01 (0.98-1.05)	0.479
Model S2	Choline (per 1 SD/day = 90 mg/day)	13,320	4,492,760	1.01 (0.98-1.04)	0.394
Model S2	Betaine (per 1 SD/day = 88 mg/day)	13,320	4,492,760	0.92 (0.79-1.07)	0.251
Model S2	Methionine (per 1g/day)	13,320	4,492,760	0.96 (0.89-1.03)	0.278
Model S3	MGD score	13,320	4,492,760	0.99 (0.98-1.01)	0.311
Model S3	Dietary folate (per 1 SD/day = 128 µg/day)	13,320	4,492,760	1.00 (0.97-1.03)	0.962
Model S3	Choline (per 1 SD/day = 90 mg/day)	13,320	4,492,760	1.00 (0.97-1.03)	0.962
Model S3	Betaine (per 1 SD/day = 88 mg/day)	13,320	4,492,760	0.99 (0.96-1.02)	0.422
Model S3	Methionine (per 1g/day)	13,320	4,492,760	0.97 (0.92-1.02	0.233
Model S4	MGD score	13,320	4,492,760	0.99 (0.98-1.01)	0.393
Model S4	Dietary folate (per 1 SD/day = 128 µg/day)	13,320	4,492,760	1.02 (0.99-1.06)	0.204
Model S4	Choline (per 1 SD/day = 90 mg/day)	13,320	4,492,760	1.00 (0.98-1.03)	0.772
Model S4	Betaine (per 1 SD/day = 88 mg/day)	13,320	4,492,760	0.98 (0.95-1.01)	0.212
Model S4	Methionine (per 1g/day)	13,320	4,492,760	0.98 (0.95-1.01)	0.254
Model S5	MGD score	10,766	3,612,534	0.99 (0.98-1.01)	0.427
Model S5	Dietary folate (per 1 SD/day = 128 µg/day)	10,766	3,612,534	1.01 (0.97-1.05)	0.577
Model S5	Choline (per 1 SD/day = 90 mg/day)	10,766	3,612,534	1.00 (0.98-1.03)	0.772
Model S5	Betaine (per 1 SD/day = 88 mg/day)	10,766	3,612,534	0.98 (0.95-1.01)	0.212
Model S5	Methionine (per 1g/day)	10,766	3,612,534	0.98 (0.95-1.01)	0.254
Model S6	MGD score	13,320	4,492,760	1.00 (0.98-1.01)	0.492
Model S6	Dietary folate (per 1 SD/day = 128 µg/day)	13,320	4,492,760	1.01 (0.99-1.03)	0.512
Model S6	Choline (per 1 SD/day = 90 mg/day)	13,320	4,492,760	1.00 (0.98-1.02)	0.891
Model S6	Betaine (per 1 SD/day = 88 mg/day)	13,320	4,492,760	0.99 (0.98-1.01)	0.433
Model S6	Methionine (per 1g/day)	13,320	4,492,760	0.97 (0.92-1.02)	0.188
Model S7	MGD score	13,320	4,492,760	1.00 (0.98-1.01)	0.438
Model S7	Dietary folate (per 1 SD/day =128 µg/day)	13,320	4,492,760	1.01 (0.98-1.05)	0.558
Model S7	Choline (per 1 SD/day = 90 mg/day)	13,320	4,492,760	1.00 (0.98-1.03)	0.957
Model S7	Betaine (per 1 SD/day = 88 mg/day)	13,320	4,492,760	0.99 (0.96-1.01)	0.412
Model S7	Methionine (per 1g/day)	13,320	4,492,760	0.97 (0.92-1.02)	0.181
Model S8	MGD score	13,320	4,492,760	1.00 (0.98-1.01)	0.430
Model S8	Dietary folate (per 1 SD/day =128 µg/day)	13,320	4,492,760	1.01 (0.98-1.05)	0.560
Model S8	Choline (per 1 SD/day = 90 mg/day)	13,320	4,492,760	1.00 (0.98-1.03)	0.939
Model S8	Betaine (per 1 SD/day = 88 mg/day)	13,320	4,492,760	0.99 (0.96-1.01)	0.367
Model S8	Methionine (per 1g/day)	13,320	4,492,760	0.98 (0.96-1.01)	0.193
Model S9	MGD score	13,320	4,492,760	1.00 (0.99-1.00)	0.220

Model S10	MGD score	13,320	4,492,760	1.00 (0.99-1.00)	0.260

Model a: fully adjusted model (see Table 3 in the main text)
Model S1: as model a, but additionally adjusted for dietary intakes of vitamin B12 (continuous), vitamin B6 (continuous) and vitamin B2 (continuous)
Model S2: as model a, but additionally adjusted for dietary intakes of serine (continuous) and glycine (continuous)
Model S3: as model a, but additionally adjusted for the Mediterranean Diet Score (continuous), and no longer adjusted for dietary fibre intake
Model S4: as model a, but additionally adjusted for dietary intakes of the food groups fruit (g/day, continuous), vegetables (g/day, continuous), cereal (g/day, continuous), dairy and dairy products (g/day, continuous), meat and meat products (g/day, continuous), and no longer adjusted for dietary fibre intake
Model S5: excluding participants with missing values in any of the covariates of model a
Model S6: as model a, but adjusted for energy intake using the residual method instead of the standard method: center-specific residuals for dietary folate, choline, betaine and methionine intake were added to center-specific mean values to maintain geographical specificity of dietary assessments and to recuperate the original scale for easier interpretation respectively
Model S7: as model a, but adjusted for alcohol free energy intake (kcal/day) using the standard method instead of adjusted for total energy intake (kcal/day)
Model S8: as model a, but analyses were stratified by five-year categories of age at recruitment, instead of one-year categories
Model S9: an additional MGD-score was obtained by adding the z-scores of the individual folate, choline, betaine, methionine, vitamin B12, B6 and B2 intakes to account for their overall association, including co-factors within the one-carbon metabolism, with BC risk.
Model S10: an additional MGD-score was obtained by adding the z-scores of the individual folate, choline, betaine, methionine, vitamin B12, B6, B2, serine and glycine intakes to account for their overall association, including co-factors within the one-carbon metabolism and amino-acids containing methyl groups, with BC risk.
Abbreviations: HR: hazard ratio; CI: confidence interval; BC: breast cancer; MGD: methyl-group donor; N: number of participants; SD: standard deviation

APPENDIX B - Dietary methyl-group donor intake and breast cancer risk: European Prospective Investigation into Cancer and Nutrition (EPIC)

Appendix B – Table S2: Proportion of contributions of each food group to the total dietary intakes of folate, choline, betaine and methionine among 318,686 women in the European Prospective Investigation into Cancer and Nutrition (EPIC) cohort

**Food groups**	**Source**	**Number of consumers**	**Betaine (%)**	**Choline (%)**	**Methionine (%)**	**Folate (%)**
Potatoes and other tubers	Plant	314,704	0.1	13.6 (#3)	1.5	2.2
Vegetables	Plant	318,629	4.4 (#2)	9.4	3.1	21.5 (#2)
Legumes	Plant	239,649	0.0	1.4	0.9	5.7
Fruits, nuts and seeds	Plant	317,549	0.3	6.1	1.7	10.2 (#3)
Dairy products	Animal	318,408	1.8	16.9 (#1)	37.7 (#1)	8.9
Cereal and cereal products	Plant	318,597	88.9 (#1)	10.2	8.6	29.9 (#1)
Meat and meat products	Animal	317,816	2.3 (#3)	12.4	24.1 (#2)	2.9
Fish and shellfish	Animal	301,325	0.3	8.0	13.9 (#3)	1.0
Egg and egg products	Animal	312,790	0.1	16.4 (#2)	4.2	2.1
Fat	Mixed	318,658	0.0	0.8	0.1	0.1
Sugar and confectionery	Mixed	309,980	0.1	1.7	0.3	0.8
Cake and biscuits	Mixed	304,723	0.6	3.5	2.4	3.1
Non-alcoholic beverages	Mixed	317,395	0.0	4.7	0.2	8.2
Alcoholic beverages	Plant	273,719	0.1	2.3	0.0	0.8
Condiments and sauces	Mixed	307,555	0.1	0.6	0.4	0.5
Soups, bouillons	Mixed	261,306	0.8	1.1	0.5	1.0
Miscellaneous	Mixed	133,528	0.1	0.6	0.4	0.5

#1: Most important food group that contributes to the total dietary intake of betaine, choline, methionine or folate, respectively, according to the columns
#2: Second most important food group that contributes to the total dietary intake of betaine, choline, methionine or folate, respectively, according to the columns
#3: Third most important food group that contributes to the total dietary intake of betaine, choline, methionine or folate, respectively, according to the columns

APPENDIX C - Dietary methyl-group donor intake and breast cancer risk: European Prospective Investigation into Cancer and Nutrition (EPIC)

Appendix C - Table S3: Characteristics of women in the European Prospective Investigation into Cancer and Nutrition (EPIC) cohort (*N* = 318,686) by lowest, middle and highest quintiles of the methyl-group donor score (MGD-score).

	**Methyl-group donor score**		
**Characteristics**	**Q1**	**Q3**	**Q5**
Number of participants	63,737	63,737	63,738
Number of cases	2568	2700	2795
Energy intake (kcal/day) [a]	1430.2 (301.9)	1950.7 (318.7)	2672.1 (457.4)
Fibre intake (g/day) [a]	16.6 (5.2)	22.9 (6.2)	30.9 (9.3)
Alcohol intake (g/day) [a]	6.7 (10.6)	7.8 (11.1)	10.1 (13.6)
Vegetable intake (g/day) [b]	129.4 (74.5; 165.4)	179.2 (122.6; 247.5)	274.8 (191.4; 380.1)
Fruit intake (g/day) [b]	170.3 (80.8; 230.3)	212.9 (129.1; 317.5)	289.5 (186.8; 420.8)
Cereal intake (g/day) [b]	141.1 (98.5; 174.2)	186.5 (142.4; 236.1)	275.7 (209.1; 356.4)
Dairy intake (g/day) [b]	208.7 (99.2; 283.0)	304.7 (188.6; 451.7)	381.2 (242.6; 564.3)
Meat intake (g/day) [b]	62.5 (38.4; 84.5)	86.4 (56.7; 116.0)	114.9 (75.8; 155.0)
Fish and Shellfish (g/day) [b]	17.2 (8.1; 31.9)	29.6 (14.8; 52.5)	40.7 (22.9; 67.8)
Egg and egg products (g/day) [b]	7.6 (2.5; 14.0)	14.5 (7.4; 22.8)	22.9 (13.1; 31.2)
Age at recruitment (years) [a]	50.5 (9.9)	50.8 (9.8)	50.7 (9.1)
Height (cm) [a]	162.6 (6.5)	162.7 (6.6)	162.1 (6.5)
Weight (kg) [a]	65.8 (11.6)	65.6 (11.6)	64.9 (11.6)
BMI (kg/m^2) [a]	24.9 (4.3)	24.8 (4.3)	24.7 (4.4)
Waist-to-hip ratio [a]	0.8 (0.01)	0.8 (0.1)	0.8 (0.1)
Age at menopause (years) [a]	48.7 (4.8)	48.7 (5.0)	48.7 (5.0)
Menopausal status at recruitment [c]			
Premenopausal (%)	34	35	36
Postmenopausal (%)	47	46	43
Perimenopausal (%)	19	19	21
Age at menarche [c]			
≤ 12 years (%)	31	35	40
13 years (%)	31	28	27
14 years (%)	21	21	20
≥ 15 years (%)	17	15	13
And age at first full term pregnancy [c]			
Nulliparous (%)	16	15	12
≤ 21 years (%)	21	17	16
22 – 29 years (%)	46	53	56
≥ 30 years (%)	9	11	12
Unknown (%)	7	3	4
Ever breastfed [c]			

Appendix C - Table S3: Characteristics of women in the European Prospective Investigation into Cancer and Nutrition (EPIC) cohort (*N* = 318,686) by lowest, middle and highest quintiles of the methyl-group donor score (MGD-score).

	Methyl-group donor score		
**Characteristics**	**Q1**	**Q3**	**Q5**
No (%)	25	25	26
Yes (%)	58	65	66
Unknown (%)	18	10	8
First degree relative (mother or sister) diagnosed with breast cancer [c]			
No (%)	25	45	51
Yes (%)	3	4	5
Unknown (%)	72	51	44
Smoking status [c]			
Never (%)	52	55	59
Former (%)	23	24	22
Current (%)	23	19	16
Unknown (%)	2	2	3
Physical activity [c]			
Inactive (%)	17	14	13
Moderately inactive (%)	29	34	42
Moderately active (%)	34	40	36
Active (%)	6	8	8
Unknown (%)	14	4	1
Education level [c]			
No schooling/ primary (%)	30	26	25
Technical/ professional/ secondary (%)	48	47	43
Higher education/ University (%)	20	23	27
Unknown (%)	3	4	5
Ever use of contraceptive pill [c]			
No (%)	34	39	41
Yes (%)	60	59	58
Unknown (%)	6	2	1
Ever use of hormone replacement therapy [c]			
No (%)	62	69	73
Yes (%)	26	25	24
Unknown (%)	13	6	3
Ever use of dietary supplements [c]			
No (%)	44	50	58
Yes (%)	36	37	33
Unknown (%)	21	12	10

[a] Mean (SD) for continuous variables

Appendix C - Table S3: Characteristics of women in the European Prospective Investigation into Cancer and Nutrition (EPIC) cohort (*N* = 318,686) by lowest, middle and highest quintiles of the methyl-group donor score (MGD-score).

	Methyl-group donor score		
**Characteristics**	**Q1**	**Q3**	**Q5**

[b] Median (25th percentile; 75th percentile) for continuous variables

[c] Frequency (%) for categorical variables

Abbreviations: EPIC: European Prospective Investigation into Cancer and nutrition; Q: quintile; SD: standard deviation; N: number of participants; BMI: Body Mass Index; MGD: methyl-group donor

APPENDIX D - Dietary methyl-group donor intake and breast cancer risk: European Prospective Investigation into Cancer and Nutrition (EPIC)

Appendix D - Table S4: Hazard ratios (HRs) and 95% confidence intervals (CIs) for breast cancer (BC) by daily dietary intake of methyl-group donors for **oestrogen receptor (ER) positive and negative tumors** in women in the EPIC cohort

	**ER positive breast cancers** (*N* = 327,931)				**ER negative breast cancers** (*N* = 322,055)				
	N of BC cases	Person years	Multivariable model[a] HR (95% CI)	$P_{continuous}$	N of BC cases	Person years	Multivariable model[a] HR (95% CI)	$P_{continuous}$	$P_{heterogeneity}$
MGD score									
Continuous (1 unit)	7513	4,446,754	1.00 (0.98-1.01)	0.594	1686	4,397,387	0.98 (0.95-1.01)	0.250	0.204
Q1: ≤ -2.58	1415	875,837	1 (Ref.)		309	866,303	1 (Ref.)		
Q2: > -2.58 – -1.00	1382	88,8456	0.97 (0.90-1.06)		307	879,446	0.99 (0.83-1.17)		
Q3: > -1.00 – 0.39	1420	895,852	0.98 (0.89-1.07)		351	886,888	1.07 (0.89-1.29)		
Q4: > 0.39 – 2.31	1518	900,647	0.98 (0.89-1.09)		330	890,603	0.91 (0.73-1.12)		
Q5: > 2.31	1778	885,962	1.03 (0.91-1.17)		389	874,147	0.87 (0.66-1.14)		
P_{trend}			0.482				0.185		
Dietary folate (µg/day)									
Continuous (per 1 SD =128 µg/day)	7513	4,446,754	1.01 (0.97-1.06)	0.556	1686	4,397,387	0.91 (0.83-1.01)	0.082	0.184
Q1: ≤ 242	1312	891,414	1 (Ref.)		284	882,647	1 (Ref.)		
Q2: > 242 - 298	1385	890,517	0.93 (0.86-1.01)		313	881,632	0.94 (0.79-1.12)		
Q3: > 298 - 357	1466	890,828	0.92 (0.84-1.01)		335	881,168	0.90 (0.75-1.09)		
Q4: > 357 - 440	1628	888,475	0.97 (0.88-1.08)		363	877,858	0.87 (0.70-1.07)		
Q5: > 440	1722	885,522	0.99 (0.87-1.13)		391	874,083	0.81 (0.62-1.06)		
P_{trend}			0.567				0.123		
Dietary choline (mg/day)									
Continuous (per 1 SD = 90 mg/day)	7513	4,446,754	1.00 (0.97-1.03)	0.861	1686	4,397,387	1.01 (0.95-1.08)	0.760	0.725
Q1: ≤ 202	1399	872,991	1 (Ref.)		318	863,802	1 (Ref.)		
Q2: > 202 - 244	1452	884,695	1.01 (0.93-1.09)		342	875,234	1.07 (0.91-1.26)		
Q3: > 244 - 285	1431	895,804	0.97 (0.89-1.05)		294	886,100	0.89 (0.75-1.06)		
Q4: > 285 - 339	1497	900,079	0.97 (0.89-1.06)		329	890,092	0.94 (0.78-1.13)		
Q5: > 339	1734	893,184	1.01 (0.91-1.12)		403	882,158	0.98 (0.79-1.21)		
P_{trend}			0.938				0.632		

Dietary betaine (mg/day)									
Continuous (per 1 SD = 88 mg/day)	7513	4,446,754	0.99 (0.95-1.02)	0.411	1686	4,397,387	0.98 (0.92-1.05)	0.638	0.945
Q1: ≤ 39	1628	830,958	1 (Ref.)		381	820,871	1 (Ref.)		
Q2: > 39 - 88	1497	924,908	0.96 (0.88-1.04)		339	915,138	0.95 (0.80-1.13)		
Q3: > 88 - 132	1449	897,150	1.00 (0.90-1.10)		301	887,260	0.95 (0.78-1.17)		
Q4: > 132 - 193	1528	901,167	0.99 (0.89-1.10)		347	890,824	1.03 (0.83-1.27)		
Q5: > 193	1411	892,572	0.95 (0.85-1.06)		318	883,294	0.97 (0.77-1.22)		
P_{trend}			0.581				0.912		
Dietary methionine (g/day)									
Continuous (per 1g/day)	7513	4,446,754	0.98 (0.92-1.05)	0.582	1686	4,397,387	0.92 (0.80-1.07)	0.278	0.455
Q1: ≤ 1.06	1242	903,007	1 (Ref.)		261	894,555	1 (Ref.)		
Q2: > 1.06 – 1.30	1458	887,242	1.05 (0.97-1.14)		317	877,117	1.10 (0.93-1.30)		
Q3: > 1.30 – 1.54	1494	889,372	1.02 (0.94-1.11)		366	879,904	1.20 (1.01-1.42)		
Q4: > 1.54 – 1.85	1551	888,050	1.00 (0.92-1.09)		357	878,057	1.08 (0.90-1.31)		
Q5: > 1.85	1768	879,083	1.02 (0.92-1.13)		385	867,754	1.00 (0.80-1.25)		
P_{trend}			0.939				0.662		

[a] Multivariable model: Cox regression model stratified by study center and one year categories of age at recruitment and adjusted for total energy intake (kcal/day), alcohol intake (g/day), dietary fibre intake (g/day), height (cm), BMI (kg/m^2), highest level of education (primary/no schooling, technical/professional/secondary, longer education, unknown), physical activity (inactive, moderately inactive, moderately active, active, unknown), smoking status (never, former, current smoker, unknown), ever use of vitamin/mineral supplements (yes, no, unknown), menopausal status at recruitment (premenopausal, postmenopausal (including surgical postmenopausal), perimenopausal), age at menarche (≤ 12, 13, 14, ≥ 15), ever use of hormones for menopause (no, yes, unknown), ever use of contraceptive pill (no, yes, unknown), and age at first full term pregnancy (nulliparous, ≤ 21y, 22-29y, ≥30y).

Abbreviations: HR: hazard ratio; CI: confidence interval; BC: breast cancer; Q: quintile; Ref: reference; MGD: methyl-group donor; N: number of participants; SD: standard deviation

**Appendix D - Table S5:** Hazard ratios (HRs) and 95% confidence intervals (CIs) for breast cancer (BC) by daily dietary intake of methyl-group donors for **progesterone receptor (PR) positive and negative tumors** in women in the EPIC cohort									
	**PR positive breast cancers** (*N* = 325,488)				**PR negative breast cancers** (*N* = 323,001)				
	N of BC cases	Person years	Multivariable model[a] HR (95% CI)	$P_{continuous}$	N of BC cases	Person years	Multivariable model[a] HR (95% CI)	$P_{continuous}$	$P_{heterogeneity}$
MGD score									
Continuous (1 unit)	5083	4,427,537	1.00 (0.98-1.02)	0.816	2620	4,406,125	0.99 (0.96-1.02)	0.387	0.572
Q1: ≤ -2.58	982	872,424	1 (Ref.)		461	867,749	1 (Ref.)		
Q2: > -2.58 – -1.00	901	884,826	0.97 (0.88-1.07)		452	880,757	0.97 (0.84-1.11)		
Q3: > -1.00 – 0.39	938	892,105	0.99 (0.88-1.10)		539	888,717	1.08 (0.93-1.26)		
Q4: > 0.39 – 2.31	999	896,380	0.98 (0.86-1.11)		521	892,441	0.95 (0.80-1.13)		
Q5: > 2.31	1263	881,802	1.05 (0.90-1.23)		647	876,460	0.97 (0.78-1.21)		
P_{trend}			0.405				0.711		
Dietary folate (µg/day)									
Continuous (per 1 SD =128 µg/day)	5083	4,427,537	1.02 (0.96-1.08)	0.481	2620	4,406,125	0.96 (0.88-1.04)	0.307	0.215
Q1: ≤ 242	907	888,251	1 (Ref.)		436	884,073	1 (Ref.)		
Q2: > 242 - 298	904	886,820	0.91 (0.82-1.00)		469	883,108	0.99 (0.86-1.13)		
Q3: > 298 - 357	968	886,944	0.90 (0.80-1.00)		501	882,719	0.97 (0.83-1.13)		
Q4: > 357 - 440	1095	884,325	0.95 (0.84-1.08)		578	879,892	1.03 (0.87-1.23)		
Q5: > 440	1209	881,198	0.96 (0.82-1.12)		636	876,334	1.02 (0.82-1.27)		
P_{trend}			0.845				0.690		
Dietary choline (mg/day)									
Continuous (per 1 SD = 90 mg/day)	5083	4,427,537	0.99 (0.95-1.03)	0.529	2620	4,406,125	1.02 (0.97-1.08)	0.456	0.330
Q1: ≤ 202	1044	870,139	1 (Ref.)		502	865,353	1 (Ref.)		
Q2: > 202 - 244	1038	881,483	1.02 (0.93-1.12)		507	876,746	1.01 (0.89-1.15)		
Q3: > 244 - 285	953	891,994	0.93 (0.84-1.03)		470	887,834	0.93 (0.81-1.07)		
Q4: > 285 - 339	954	895,685	0.92 (0.83-1.03)		523	891,965	1.01 (0.87-1.17)		
Q5: > 339	1094	888,236	0.97 (0.86-1.10)		618	884,227	1.08 (0.91-1.28)		
P_{trend}			0.459				0.298		
Dietary betaine (mg/day)									
Continuous (per 1 SD = 88 mg/day)	5083	4,427,537	1.00 (0.96-1.04)	0.9150	2620	4,406,125	0.98 (0.93-1.03)	0.434	0.486
Q1: ≤ 39	1050	826,876	1 (Ref.)		508	822,205	1 (Ref.)		
Q2: > 39 - 88	906	920,359	1.02 (0.91-1.15)		506	916,777	0.96 (0.83-1.11)		

Q3: > 88 - 132	989	893,397	1.10 (0.97-1.25)		502	889,089	0.92 (0.78-1.09)		
Q4: > 132 - 193	1092	897,398	1.11 (0.97-1.26)		559	892,777	0.94 (0.79-1.11)		
Q5: > 193	1046	889,506	1.07 (0.93-1.23)		545	885,277	0.93 (0.77-1.12)		
P_{trend}			0.377				0.573		
Dietary methionine (g/day)									
Continuous (per 1g/day)	5083	4,427,537	0.98 (0.90-1.06)	0.456	2620	4,406,125	0.94 (0.84-1.05)	0.275	0.359
Q1: ≤ 1.06	818	899,537	1 (Ref.)		410	895,969	1 (Ref.)		
Q2: > 1.06 – 1.30	984	883,344	1.07 (0.97-1.18)		473	878,645	1.01 (0.88-1.15)		
Q3: > 1.30 – 1.54	976	885,465	1.00 (0.90-1.10)		544	881,490	1.08 (0.94-1.24)		
Q4: > 1.54 – 1.85	1049	884,050	1.00 (0.90-1.11)		561	880,048	1.01 (0.87-1.17)		
Q5: > 1.85	1256	875,142	1.02 (0.90-1.15)		632	869,974	0.95 (0.80-1.14)		
P_{trend}			0.851				0.490		

[a] Multivariable model: Cox regression model stratified by study center and one year categories of age at recruitment and adjusted for total energy intake (kcal/day), alcohol intake (g/day), dietary fibre intake (g/day), height (cm), BMI (kg/m^2), highest level of education (primary/no schooling, technical/professional/secondary, longer education, unknown), physical activity (inactive, moderately inactive, moderately active, active, unknown), smoking status (never, former, current smoker, unknown), ever use of vitamin/mineral supplements (yes, no, unknown), menopausal status at recruitment (premenopausal, postmenopausal (including surgical postmenopausal), perimenopausal), age at menarche (≤ 12, 13, 14, ≥ 15), ever use of hormone for menopause (no, yes, unknown), ever use of contraceptive pill (no, yes, unknown), and age at first full term pregnancy (nulliparous, ≤ 21y, 22-29y, ≥30y).

Abbreviations: HR: hazard ratio; CI: confidence interval; BC: breast cancer; Q: quintile; Ref: reference; MGD: methyl-group donor

**Appendix D - Table S6:** Hazard ratios (HRs) and 95% confidence intervals (CIs) for breast cancer (BC) by daily dietary intake of methyl-group donors for **human epidermal growth factor receptor (HER2) positive and negative tumors** in women in the EPIC cohort									
	**HER2 positive breast cancers** (*N* = 321,238)				**HER2 negative breast cancers** (*N* = 323,996)				
	N of BC cases	Person years	Multivariable model[a] HR (95% CI)	P_{cont} P_{trend}	N of BC cases	Person years	Multivariable model[a] HR (95% CI)	P_{cont}	$P_{heterogeneity}$
MGD score									
Continuous (1 unit)	859	4,393,558	0.99 (0.95-1.04)	0.768	3600	4,423,226	0.99 (0.97-1.01)	0.400	0.803
Q1: ≤ -2.58	161	865,581	1 (Ref.)		833	872,490	1 (Ref.)		
Q2: > -2.58 – -1.00	170	878,815	1.15 (0.91-1.46)		696	884,452	0.95 (0.85-1.06)		
Q3: > -1.00 – 0.39	167	886,001	1.15 (0.88-1.50)		666	891,456	0.93 (0.82-1.05)		
Q4: > 0.39 – 2.31	157	889,689	1.03 (0.76-1.39)		676	895,572	0.91 (0.79-1.05)		
Q5: > 2.31	204	873,472	1.18 (0.81-1.73)		729	879,257	0.91 (0.75-1.10)		
P_{trend}			0.635				0.344		
Dietary folate (µg/day)									
Continuous (per 1 SD =128 µg/day)	859	4,393,558	0.97 (0.84-1.11)	0.640	3600	4,423,226	1.04 (0.97-1.11)	0.232	0.348
Q1: ≤ 242	117	881,718	1 (Ref.)		633	887,167	1 (Ref.)		
Q2: > 242 - 298	174	880,968	1.22 (0.95-1.56)		717	886,806	0.96 (0.85-1.07)		
Q3: > 298 - 357	189	880,492	1.22 (0.93-1.59)		786	886,978	1.00 (0.88-1.14)		
Q4: > 357 - 440	183	877,100	1.15 (0.85-1.57)		744	883,175	0.98 (0.85-1.13)		
Q5: > 440	196	873,280	1.28 (0.87-1.88)		720	879,100	1.05 (0.87-1.26)		
P_{trend}			0.407				0.519		
Dietary choline (mg/day)									
Continuous (per 1 SD = 90 mg/day)	859	4,393,558	1.03 (0.93-1.13)	0.607	3600	4,423,226	0.97 (0.92-1.02)	0.242	0.320
Q1: ≤ 202	173	863,016	1 (Ref.)		825	869,551	1 (Ref.)		
Q2: > 202 - 244	179	874,435	1.10 (0.88-1.37)		752	880,598	1.02 (0.91-1.13)		
Q3: > 244 - 285	159	885,674	0.99 (0.78-1.27)		628	890,722	0.87 (0.78-0.98)		
Q4: > 285 - 339	155	889,251	0.96 (0.74-1.26)		674	895,168	0.94 (0.83-1.07)		
Q5: > 339	193	881,183	1.11 (0.82-1.50)		721	887,188	0.95 (0.81-1.10)		
P_{trend}			0.700				0.403		
Dietary betaine (mg/day)									
Continuous (per 1 SD = 88 mg/day)	859	4,393,558	0.98 (0.89-1.08)	0.707	3600	4,423,226	1.00 (0.95-1.05)	0.868	0.795
Q1: ≤ 39	230	820,383	1 (Ref.)		989	827,989	1 (Ref.)		
Q2: > 39 - 88	156	914,240	0.87 (0.68-1.12)		717	920,483	1.03 (0.91-1.16)		

Q3: > 88 - 132	148	886,562	1.03 (0.77-1.37)		631	891977	1.14 (0.99-1.31)		
Q4: > 132 - 193	165	889,808	0.98 (0.72-1.33)		688	895680	1.09 (0.94-1.27)		
Q5: > 193	160	882,566	0.96 (0.69-1.33)		575	887096	1.03 (0.87-1.21)		
P_{trend}			0.901				0.997		
Dietary methionine (g/day)									
Continuous (per 1g/day)	859	4,393,558	0.96 (0.78-1.17)	0.667	3600	4,423,226	0.93 (0.84-1.03)	0.176	0.259
Q1: ≤ 1.06	128	893,748	1 (Ref.)		654	899,461	1 (Ref.)		
Q2: > 1.06 – 1.30	178	876,629	1.27 (1.01-1.61)		765	882,822	1.10 (0.99-1.23)		
Q3: > 1.30 – 1.54	174	878,926	1.17 (0.91-1.51)		708	884,839	0.98 (0.87-1.11)		
Q4: > 1.54 – 1.85	173	877,111	1.07 (0.82-1.40)		715	883,132	0.96 (0.85-1.09)		
Q5: > 1.85	206	867,144	1.09 (0.80-1.49)		758	872,973	0.93 (0.79-1.08)		
P_{trend}			0.856				0.096		

[a] Multivariable model: Cox regression model stratified by study center and one year categories of age at recruitment and adjusted for total energy intake (kcal/day), alcohol intake (g/day), dietary fibre intake (g/day), height (cm), BMI (kg/m^2), highest level of education (primary/no schooling, technical/professional/secondary, longer education, unknown), physical activity (inactive, moderately inactive, moderately active, active, unknown), smoking status (never, former, current smoker, unknown), ever use of vitamin/mineral supplements (yes, no, unknown), menopausal status at recruitment (premenopausal, postmenopausal (including surgical postmenopausal), perimenopausal), age at menarche (≤ 12, 13, 14, ≥ 15), ever use of hormones for menopause (no, yes, unknown), ever use of contraceptive pill (no, yes, unknown), and age at first full term pregnancy (nulliparous, ≤ 21y, 22-29y, ≥30y).

Abbreviations: HR: hazard ratio; CI: confidence interval; BC: breast cancer; Q: quintile; Ref: reference; MGD: methyl-group donor; N: number of participants; SD: standard deviation

Appendix D - Table S7: Hazard ratios (HRs) and 95% confidence intervals (CIs) for breast cancer (BC) by daily dietary intake of methyl-group donors for **oestrogen receptor and progesterone receptor positive or negative tumors** in women in the EPIC cohort

	**ER/PR positive breast cancers** (*N* = 325,236)				**ER/PR negative breast cancers** (*N* = 321,644)				
	N of BC cases	Person years	Multivariable model[a] HR (95% CI)	$P_{continuous}$	N of BC cases	Person years	Multivariable model[a] HR (95% CI)	$P_{continuous}$	$P_{heterogeneity}$
MGD score									
Continuous (1 unit)	4833	4,426,138	1.00 (0.98-1.02)	0.777	1276	4,395,076	0.98 (0.94-1.02)	0.240	0.309
Q1: ≤ -2.58	943	872,248	1 (Ref.)		245	866,008	1 (Ref.)		
Q2: > -2.58 – -1.00	858	884,535	0.96 (0.87-1.06)		226	878,940	0.94 (0.78-1.15)		
Q3: > -1.00 – 0.39	881	891,780	0.96 (0.86-1.08)		258	886,394	1.03 (0.83-1.27)		
Q4: > 0.39 – 2.31	950	896,144	0.97 (0.86-1.11)		251	890,147	0.90 (0.71-1.16)		
Q5: > 2.31	1201	881,430	1.06 (0.90-1.24)		296	873,587	0.86 (0.63-1.18)		
P_{trend}			0.342				0.313		
Dietary folate (µg/day)									
Continuous (per 1 SD =128 µg/day)	4833	4,426,138	1.02 (0.96-1.08)	0.498	1276	4,395,076	0.91 (0.81-1.02)	0.100	0.076
Q1: ≤ 242	867	888,042	1 (Ref.)		214	882,295	1 (Ref.)		
Q2: > 242 - 298	861	886,563	0.90 (0.81-1.00)		240	881,225	0.99 (0.81-1.20)		
Q3: > 298 - 357	924	886,680	0.89 (0.80-1.00)		251	880,655	0.94 (0.75-1.16)		
Q4: > 357 - 440	1037	884,036	0.95 (0.84-1.08)		275	877,385	0.92 (0.72-1.18)		
Q5: > 440	1144	880,817	0.96 (0.82-1.13)		296	873,516	0.86 (0.63-1.17)		
P_{trend}			0.801				0.294		
Dietary choline (mg/day)									
Continuous (per 1 SD = 90 mg/day)	4833	4,426,138	0.98 (0.94-1.03)	0.459	1276	4,395,076	1.01 (0.93-1.09)	0.841	0.512
Q1: ≤ 202	995	869,860	1 (Ref.)		256	863,442	1 (Ref.)		
Q2: > 202 - 244	974	881,109	1.00 (0.91-1.10)		257	874,758	1.04 (0.87-1.25)		
Q3: > 244 - 285	911	891,781	0.93 (0.84-1.03)		214	885,674	0.85 (0.69-1.04)		
Q4: > 285 - 339	915	895,488	0.93 (0.83-1.04)		252	889,677	0.96 (0.77-1.19)		
Q5: > 339	1038	887,900	0.97 (0.85-1.10)		297	881,526	0.98 (0.77-1.26)		
P_{trend}			0.515				0.868		
Dietary betaine (mg/day)									
Continuous (per 1 SD = 88 mg/day)	4833	4,426,138	1.00 (0.96-1.04)	0.878	1276	4,395,076	0.99 (0.91-1.07)	0.711	0.672
Q1: ≤ 39	1011	826,709	1 (Ref.)		287	820,470	1 (Ref.)		
Q2: > 39 - 88	857	920,083	1.01 (0.90-1.14)		251	914,607	0.99 (0.80-1.21)		

Q3: > 88 - 132	938	893,133	1.08 (0.95-1.23)		229	886,886	0.96 (0.75-1.22)		
Q4: > 132 - 193	1039	897,075	1.09 (0.96-1.25)		263	890,289	1.01 (0.79-1.30)		
Q5: > 193	988	889,137	1.05 (0.91-1.22)		246	882,824	0.97 (0.74-1.27)		
P_{trend}			0.472				0.919		
Dietary methionine (g/day)									
Continuous (per 1g/day)	4833	4,426,138	0.98 (0.90-1.07)	0.615	1276	4,395,076	0.90 (0.77-1.07)	0.239	0.244
Q1: ≤ 1.06	788	899,375	1 (Ref.)		208	894,264	1 (Ref.)		
Q2: > 1.06 – 1.30	933	883,048	1.05 (0.95-1.16)		233	876,622	1.01 (0.83-1.23)		
Q3: > 1.30 – 1.54	923	885,187	0.98 (0.88-1.09)		275	879,396	1.13 (0.93-1.38)		
Q4: > 1.54 – 1.85	992	883,697	0.98 (0.88-1.10)		267	877,558	1.02 (0.82-1.26)		
Q5: > 1.85	1197	874,831	1.02 (0.89-1.16)		293	867,237	0.96 (0.75-1.24)		
P_{trend}			0.929				0.635		

[a] Multivariable model: Cox regression model stratified by study center and one year categories of age at recruitment and adjusted for total energy intake (kcal/day), alcohol intake (g/day), dietary fibre intake (g/day), height (cm), BMI (kg/m^2), highest level of education (primary/no schooling, technical/professional/secondary, longer education, unknown), physical activity (inactive, moderately inactive, moderately active, active, unknown), smoking status (never, former, current smoker, unknown), ever use of vitamin/mineral supplements (yes, no, unknown), menopausal status at recruitment (premenopausal, postmenopausal (including surgical postmenopausal), perimenopausal), age at menarche (≤ 12, 13, 14, ≥ 15), ever use of hormones for menopause (no, yes, unknown), ever use of contraceptive pill (no, yes, unknown), and age at first full term pregnancy (nulliparous, ≤ 21y, 22-29y, ≥30y).

Abbreviations: HR: hazard ratio; CI: confidence interval; BC: breast cancer; Q: quintile; Ref: reference; MGD: methyl-group donor; N: number of participants; SD: standard deviation
